# Psychological stress-induced systemic corticosterone directly sabotages intestinal stem cells and exacerbates colitis

**DOI:** 10.1038/s41421-025-00796-y

**Published:** 2025-05-13

**Authors:** Xiaole Sheng, Lanfei Jin, Zhengrong Yao, Jiaji Gu, Longtao Zhu, Andi Huang, Junxuan Peng, Xin Xu, Xiaolong Ge, Wei Zhou, Jinghao Sheng, Zhengping Xu, Rongpan Bai

**Affiliations:** 1 Department of General Surgery, Sir Run Run Shaw Hospital and Institute of Environmental Medicine, Zhejiang University School of Medicine, Hangzhou, Zhejiang China; 2 Liangzhu Laboratory, Zhejiang University, Hangzhou, Zhejiang China; 3 Cancer Center, Zhejiang University, Hangzhou, Zhejiang China

**Keywords:** Intestinal stem cells, Mechanisms of disease

## Abstract

Psychological stress has profound impacts on the gastrointestinal tract via the brain‒gut axis. However, its effects on intestinal stem cells (ISCs) and the resulting implication for intestinal homeostasis remain poorly understood. Here, we observed a notable reduction in both the quantity and proliferative capacity of ISCs under chronic stress conditions, driven by elevated levels of corticosterone resulting from activation of the hypothalamic‒pituitary‒adrenal (HPA) axis. Mechanistically, corticosterone directly interacts with its receptor, nuclear receptor subfamily 3 group c member 1 (NR3C1), leading to increased expression of FKBP prolyl isomerase 5 (FKBP5) in ISCs. Subsequently, FKBP5 negatively regulates AKT activation by facilitating its dephosphorylation at Ser473, ultimately enhancing nuclear translocation of forkhead box O (FoxO) and inhibiting ISC proliferative activity. Consequently, ISC dysfunction contributes to the stress-driven exacerbation of DSS-induced colitis. Collectively, these findings reveal an intrinsic brain-to-gut regulatory pathway whereby psychological stress impairs ISC activity via corticosterone elevation, providing a mechanistic explanation for stress-enhanced susceptibility to colitis.

## Introduction

The gut‒brain axis describes a complex bidirectional communication network that interconnects the brain’s emotional and cognitive centers with peripheral intestinal functions^1^. This intricate connection primarily relies on the central nervous system (CNS), the autonomic nervous system (ANS), hypothalamic‒pituitary‒adrenal (HPA) axis, and the enteric nervous system (ENS)^2^. Maladaptation to stress has been identified to disrupt the operation of these systems, thus leading to the disturbances in the brain-to-gut communication^3^. Consequently, individuals experiencing psychological stress often demonstrate physiological gut dysfunctions, including alterations in motility, secretion, and intestinal permeability^4–6^, and also face an increased risk for various gastrointestinal disorders, such as inflammatory bowel disease (IBD), irritable bowel syndrome (IBS) and other functional diseases^7–9^, highlighting the wide-ranging impact of psychological stress on gut health.

Intestinal stem cells (ISCs) are highly proliferative cells that fuel the continuous renewal of the intestinal epithelium and play a critical role in maintaining intestinal homeostasis^10^. Any disturbance that compromises the activity and function of ISCs would sensitize the intestinal epithelium to other environmental stimuli, potentially predisposing individuals to gastrointestinal disorders^11^. It is widely acknowledged that the unique properties and functions of somatic stem cells render them particularly susceptible to stressors^12–17^. However, the specific effects of psychological stress on ISC behaviors and the resulting implication for intestinal homeostasis remain poorly understood. More recently, a study has reported that the stress-induced microbial dysbiosis disrupts the intestinal cell lineage commitment^18^. Additionally, Hou et al. described a diet-microbial metabolism feedforward loop that assists in maintaining ISCs during stress^19^. These findings emphasize the critical role of the gut microbiome in linking psychological stress to ISCs. Despite the involvement of microbiota as an extrinsic cue, little is known about whether and how intrinsic responses in the organism contribute to relaying psychological stress influences to ISC population.

In this study, we revealed an intrinsic brain-to-gut regulatory pathway through which psychological stress impedes ISC behaviors via the HPA axis activation. Furthermore, we elucidated that the stress-induced systemic factor, corticosterone, directly diminishes both the quantity and proliferative activity of ISCs through its receptor NR3C1. We also demonstrated that ISC dysfunction is partially responsible for the stress-driven exacerbation of colitis. These findings provide a novel mechanistic insight into the brain‒gut connection and underscore corticosterone as a pivotal mediator connecting psychological stress to ISC dysfunction, thereby increasing susceptibility to developing colitis.

## Results

### Chronic stress leads to ISC impairment

**Fig. 1:**
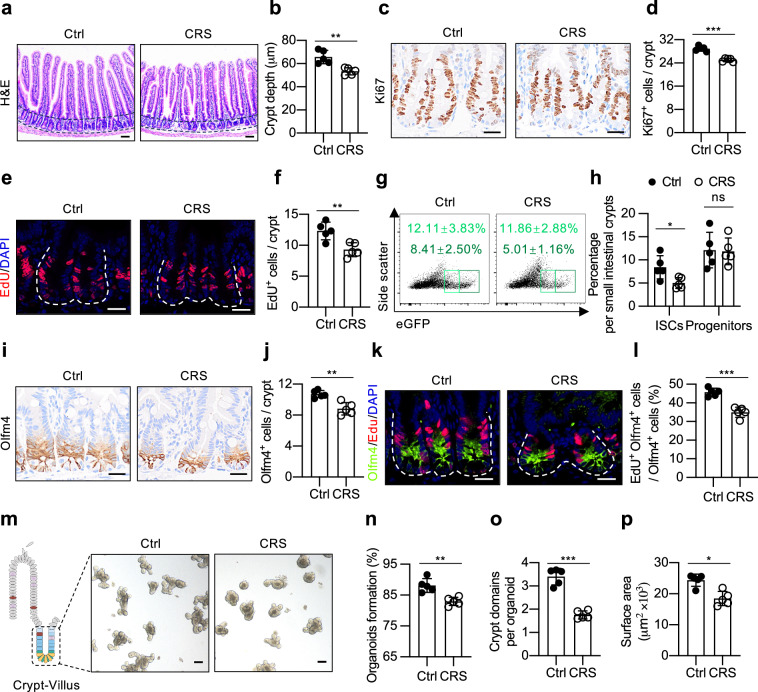
Chronic stress impairs intestinal stem cells (ISCs). **a**, **b** Hematoxylin and eosin (H&E) staining (**a**) and quantification of crypt depth (**b**) in the jejuna of control (Ctrl) and chronic restraint stress (CRS)-treated mice (*n* = 5). Scale bar: 50 µm. **c**, **d** Ki-67 staining (**c**) and quantification of Ki-67^+^ cells per crypt (**d**) in the jejuna of the Ctrl and CRS-treated mice (*n* = 5). Scale bar: 20 µm. **e**, **f** 5-Ethynyl-2’-deoxyuridine (EdU) staining after pulse labeling for 1.5 h (**e**) and quantification of EdU^+^ cells per crypt (**f**) in the jejuna of the Ctrl and CRS-treated mice (*n* = 5). Scale bar: 20 µm. **g**, **h** Representative flow cytometric plots showing Lgr5-EGFP^+^ cells (**g**) and quantification of the percentage of ISCs (Lgr5-EGFP^High^) and progenitor cells (Lgr5-EGFP^Low^) (**h**) in the small intestine of the Ctrl and CRS-treated *Lgr5-EGFP-IRES-creERT2* mice (*n* = 5). **i**, **j** Olfm4 staining (**i**) and quantification of Olfm4^+^ cells per crypt (**j**) in the jejuna of the Ctrl and CRS-treated mice (*n* = 5). Scale bar: 20 µm. **k**, **l** EdU and Olfm4 co-staining (**k**) and quantification of the proportion of EdU^+^ Olfm4^+^ cells (**l**) in the jejuna of the Ctrl and CRS-treated mice (*n* = 5). Scale bar: 20 µm. **m**‒**p** Representative images of day 4 organoids (**m**) and quantification of organoid formation (**n**), bud number (**o**) and surface area (**p**) from the Ctrl and CRS-treated mice (*n* = 5). Scale bar: 100 µm. The data are presented as the mean ± SD. The statistical analysis was performed by an unpaired two-tailed Student’s *t*-test for normally distributed data or the Mann‒Whitney test for non-normally distributed data. ns, *p* ≥ 0.05, *p < 0.05, **p < 0.01, ***p < 0.001.

To investigate the influence of psychological stress on intestinal homeostasis, we first employed a well-established mouse model of prolonged psychological stress known as chronic restraint stress (CRS) in C57BL/6 J mice (Supplementary Fig. S1a). As previously reported^20^, the mice subjected to CRS experienced significant weight loss (Supplementary Fig. S1b) and exhibited increased immobility time in the forced swimming test (FST) (Supplementary Fig. S1c), indicating depression-like behavior. To determine the impact of CRS on gut homeostasis, we subsequently examined the small intestine of these mice and observed a significant reduction in intestinal length (Supplementary Fig. S1d). Further examination of the small intestine revealed a shortened crypt depth, a mild reduction in villus height, and a notable decrease in the numbers of Ki-67- and EdU-positive cells in the intestinal crypts (Fig. 1a‒f; Supplementary Fig. S1e), demonstrating that the proliferative capacity of the intestinal cells was impaired. Along with the reduction in villus height, a slight decrease in the number of alkaline phosphatase (Alpi)^+^ enterocyte was observed in the CRS-treated mice (Supplementary Fig. S1f, g). However, CRS had no effects on the frequency of periodic acid-Schiff (PAS)^+^ secretory goblet cells, lysozyme (Lyz)^+^ Paneth cells and chromogranin A (ChgA)^+^ enteroendocrine cells per crypt-villus unit of the small intestine (Supplementary Fig. S1h‒m).

To validate these observations, we employed another stress model, chronic unpredictable stress (CUS), which similarly resulted in significant reduction in body weight, small intestinal length, crypt depth (Supplementary Fig. S2a‒e), as well as the numbers of Ki-67-positive cells (Supplementary Fig. S2f, g). In contrast to CRS, CUS exposure did not impact the numbers of Alpi^+^ enterocytes, which may be attributed to the unchanged villus length observed in CUS-treated mice (Supplementary Fig. S2h‒j). However, similar to CRS, CUS did not affect PAS^+^ secretory goblet cells, the frequency of Lyz^+^ Paneth cells, or ChgA^+^ enteroendocrine cells (Supplementary Fig. S2k‒p). These findings suggest that the chronic stress exposure minimally affects the differentiation of epithelial cell types in the small intestine.

To determine whether chronic stress affects ISCs, thus contributing to the alteration of intestinal homeostasis, we conducted CRS experiments in *Lgr5-EGFP-IRES-creERT2* knock-in mice, which enabled us to analyze Lgr5-EGFP^hi^ ISCs and their daughter, more differentiated EGFP^low^ progenitors using flow cytometry^21^. Compared to the nonstressed controls, the stressed mice exhibited an approximately 1.5-fold decrease in the frequency of Lgr5-EGFP^hi^ ISCs (5.01% ± 1.16% vs. 8.41% ± 2.50%), while no significant difference was observed in the frequency of differentiated EGFP^low^ progenitors between the two groups (Fig. 1g, h). To validate this observation, we performed direct immunofluorescence staining for GFP on intestinal tissues from these mice. Consistent with the flow cytometric results, immunostaining revealed a significant reduction in both the frequency of GFP-positive crypts and the number of GFP^+^ ISCs per crypt of CRS-exposed mice (Supplementary Fig. S3a‒c). These results were further supported by the phenotypic analysis of ISCs via immunohistochemical staining of olfactomedin 4 (Olfm4) (Fig. 1i, j; Supplementary Fig. S2q, r), another established marker coexpressed in Lgr5^+^ ISCs in the mouse small intestine^22^. Moreover, co-staining of ISCs with Ki-67 or EdU revealed pronounced inhibition of their proliferative activity in response to chronic stress (Fig. 1k, l; Supplementary Fig. S3d). Additionally, this inhibitory effect of CRS on small intestinal stem cell was also observed in colonic stem cells (Supplementary Fig. S3e‒h). Collectively, these data demonstrate that chronic stress impairs the proliferative capacity of ISCs, leading to a reduction in their number.

To further evaluate the effect of stress on ISC function, we isolated small intestinal crypts from both stressed and nonstressed mice and tested their clonal, multipotent organoid formation and budding capacities ex vivo. The results showed that, compared with those in the control group, the crypts in the stressed mice exhibited a diminished ability to form organoids (Fig. 1m, n). Moreover, the bud numbers and overall sizes of these organoids were notably decreased (Fig. 1o, p), confirming that stress impaired the activity of stem cells within crypts, as only stem cells are capable of self-renewing and differentiating into various cell types required for organoid formation and maintenance^23^. Notably, during subcloning, there was no significant difference in the generation of secondary organoids from dissociated crypt-derived primary organoids between the CRS-treated and control groups (Supplementary Fig. S3i‒l), suggesting that the stress-induced impairment in ISCs might be reversible when the stressor has been eliminated.

Taken together, these findings reveal that chronic stress can impair ISCs, thereby affecting intestinal homeostasis.

### Adrenalectomy (ADX) eliminates the effect of stress on ISCs

**Fig. 2:**
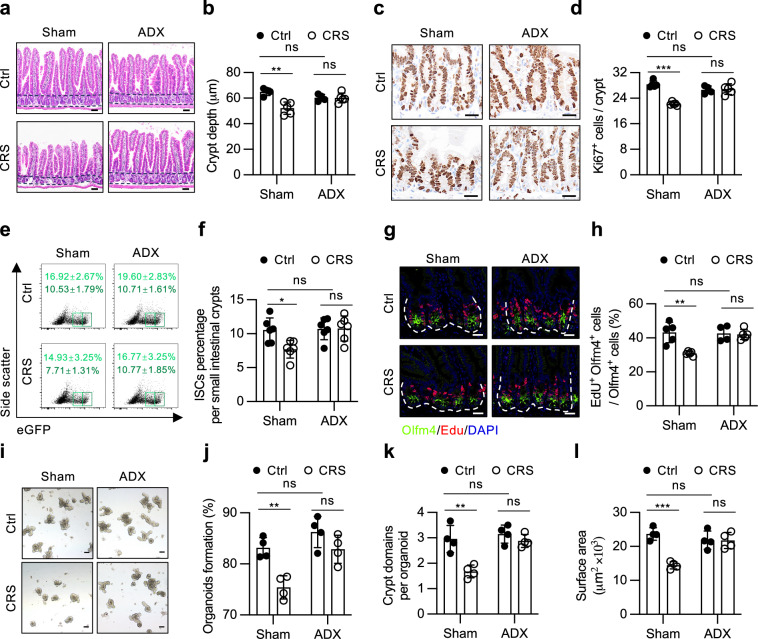
Removal of adrenal glands counteracts the effect of stress on ISCs. **a**, **b** H&E staining (**a**) and quantification of crypt depth (**b**) in the jejuna of Sham + Ctrl (*n* = 4), Sham + CRS (*n* = 5), ADX + Ctrl (*n* = 4) and ADX + CRS (*n* = 5) mice. ADX adrenalectomize. Scale bar: 50 µm. **c**, **d** Ki-67 staining (**c**) and quantification of Ki-67^+^ cells per crypt (**d**) in the jejuna of Sham + Ctrl (*n* = 5), Sham + CRS (*n* = 5), ADX + Ctrl (*n* = 4) and ADX + CRS (*n* = 5) mice. Scale bar: 20 µm. **e**, **f** Representative flow cytometric plots for Lgr5-EGFP^+^ cells (**e**) and quantification of the percentage of ISCs (Lgr5-EGFP^High^) (**f**) in the small intestine of the Sham + Ctrl, Sham + CRS, ADX + Ctrl and ADX + CRS *Lgr5-EGFP-IRES-creERT2* mice (*n* = 6). **g**, **h** EdU and Olfm4 staining (**g**) and quantification of the proportion of EdU^+^ Olfm4^+^ cells (**h**) in the jejuna of the Sham + Ctrl (*n* = 5), Sham + CRS (*n* = 5), ADX + Ctrl (*n* = 4) and ADX + CRS (*n* = 5) mice. EdU (0.2 mg/25 g body weight) was administered to mice 1.5 h prior to sacrifice. Scale bar: 20 µm. **i**‒**l** Representative images of day 4 organoids (**i**) and quantification of organoid formation (**j**), bud number (**k**) and surface area (**l**) from the Sham + Ctrl, Sham + CRS, ADX + Ctrl and ADX + CRS mice (*n* = 4). Scale bar: 100 µm. The data are presented as the mean ± SD. The statistical analysis was performed by an unpaired two-tailed Student’s *t*-test for normally distributed data or the Mann‒Whitney test for nonnormally distributed data. ns, *p* ≥ 0.05, *p < 0.05, **p < 0.01, ***p < 0.001.

One fundamental mechanism of chronic, sustained stress exposure is to activate the HPA axis or the sympathetic‒adreno-medullar (SAM) axis, which leads to the release of stress hormones or catecholamines, respectively, from the adrenal glands^24^. To investigate whether these systemic factors can transmit the effects of stress to intestinal homeostasis, we removed both adrenal glands from wild-type (WT) mice. After 1-week recovery period following surgery, these mice were subjected to CRS treatment for 3 weeks (Supplementary Fig. S4a). While adrenalectomy itself did not significantly impact the mouse weight, both the sham-operated (sham) mice and the adrenalectomized mice experienced a similar degree of weight loss during CRS exposure (Supplementary Fig. S4b). Consistent with the observations in the WT mice, CRS induced a notable reduction in intestinal length in the sham mice. However, this inhibitory effect was effectively reversed in the adrenalectomized mice (Supplementary Fig. S4c). Furthermore, ADX appeared to prevent the shortening of crypt depth, the reduction in villus height, and the decrease in Ki-67-positive cells induced by CRS treatment, as shown by morphological analyses (Fig. 2a‒d; Supplementary Fig. S4d).

To determine whether the adrenal glands participate in the detrimental effect of stress on ISCs, we further performed ADX on *Lgr5-EGFP-IRES-creERT2* knock-in mice and subjected them to CRS. Flow cytometry revealed that, under stress conditions, the reduced frequency of Lgr5-EGFP^hi^ ISCs observed in the small intestine of sham mice was roughly reversed in the ADX mice (Fig. 2e, f; Supplementary Fig. S4e). These findings were further validated by immunostaining for Olfm4 and GFP, which confirmed the recovery of ISCs number in ADX mice under stress (Supplementary Fig. S4f‒j). Importantly, the percentage of proliferative ISCs, as indicated by EdU^+^Olfm4^+^ or GFP^+^Ki-67^+^ cells, in the stressed ADX mice also returned to levels similar to those observed in nonstressed controls (Fig. 2g, h; Supplementary Fig. S4k). Furthermore, immunofluorescence staining for GFP and Ki67 in colonic sections revealed that ADX also restored both the numbers and proliferative activity of colonic stem cells, which were reduced by CRS (Supplementary Fig. S4l‒o).

Using an ex vivo organoid system, we further confirmed that ADX also effectively restored the regenerative capacity of the small intestinal crypts suppressed by stress (Fig. 2i‒l). Collectively, these data suggest that the adrenal glands mediate the detrimental effect of stress on intestinal stem cells.

### Corticosterone relays the effect of stress to ISCs

**Fig. 3:**
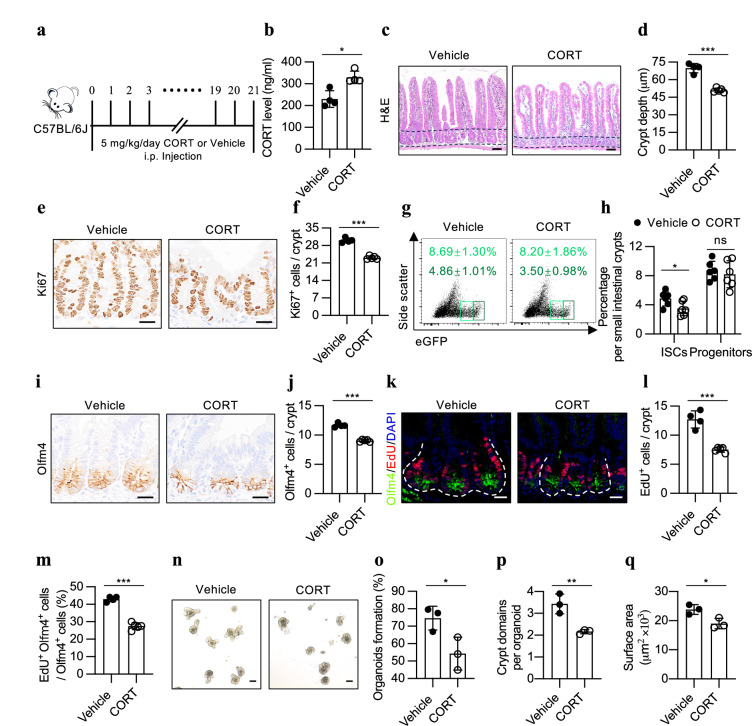
Corticosterone reproduces the effect of stress on ISCs. **a** Schematic representation of CORT administration. C57BL/6 mice were injected intraperitoneally with CORT (5 mg/kg) or vehicle once daily for 3 weeks at varying times of the day. CORT corticosterone. **b** Plasma CORT levels in mice measured 3 h after a single vehicle or CORT injection (*n* = 4). **c**, **d** H&E staining (**c**) and quantification of crypt depth (**d**) in the jejuna of the vehicle (*n* = 4) and CORT-treated (*n* = 5) mice. Scale bar: 50 µm. **e**, **f** Ki-67 staining (**e**) and quantification of Ki-67^+^ cells per crypt (**f**) in the jejuna of the vehicle (*n* = 4)- and CORT-treated (*n* = 5) mice. Scale bar: 20 µm. **g**, **h** Representative flow cytometric plots showing EGFP^+^ cells (**g**) and quantification of the percentage of ISCs (Lgr5-EGFP^High^) and progenitor cells (Lgr5-EGFP^Low^) (**h**) in the small intestine of the vehicle- and CORT-treated *Lgr5-EGFP-IRES-creERT2* mice (*n* = 6). **i**, **j** Olfm4 staining (**i**) and quantification of Olfm4^+^ cells per crypt (**j**) in the jejuna of the vehicle (*n* = 4)- and CORT (*n* = 5)-treated mice. Scale bar: 20 µm. **k**‒**m** EdU and Olfm4 co-staining (**k**), quantification of EdU^+^ cells per crypt (**l**) and the percentage of EdU^+^ Olfm4^+^ cells (**m**) in the jejuna of the vehicle- (*n* = 4) and CORT (*n* = 5)-treated mice. EdU (0.2 mg/25 g body weight) was administered to mice 1.5 h prior to sacrifice. Scale bar: 20 µm. **n**‒**q** Representative images of day 4 organoids (**n**) and quantification of organoid formation (**o**), bud number (**p**) and surface area (**q**) from the vehicle- and CORT-treated mice (*n* = 3). Scale bar: 100 µm. The data are presented as the mean ± SD. The statistical analysis was performed by an unpaired two-tailed Student’s *t*-test for normally distributed data or the Mann‒Whitney test for non-normally distributed data. ns, *p* ≥ 0.05, *p < 0.05, **p < 0.01, ***p < 0.001.

Psychological stressors activate the adrenal glands to release stress hormones and catecholamines into the bloodstream (Supplementary Fig. S5a)^13,24^. In line with this, we observed an increase in the corticosterone concentration and a trend toward increased noradrenaline levels in the blood of the CRS-exposed mice (Supplementary Fig. S5b, c). To investigate whether these hormones can mediate the effect of stress on intestinal homeostasis, we administered corticosterone or noradrenaline to WT mice via intraperitoneal injection (Fig. 3a, b; Supplementary Fig. S5d). Interestingly, only the mice treated with corticosterone, and not those treated with noradrenaline, displayed similar alterations in small intestinal homeostasis as those induced by CRS. These changes included shortened crypt depth, a notable decrease in Ki-67-positive cells, and a reduction in the numbers of Lgr5- or Olfm4-positive ISCs in the small intestinal crypts (Fig. 3c‒j; Supplementary Fig. S5e‒m). Furthermore, treatment with corticosterone not only decreased the number of small intestinal stem cells but also suppressed their proliferative capacity, as evidenced by the reduction in frequency of EdU^+^Olfm4^+^ or GFP^+^Ki-67^+^ cells (Fig. 3k‒m; Supplementary Fig. S5n). A similar effect was observed in colonic stem cells as well (Supplementary Fig. S5o‒r).

Using ex vivo organoid culture system, we further demonstrated that corticosterone treatment led to a notable decrease in the formation of organoids from isolated small intestinal crypts, as well as a reduction in the bud number and overall size of the corresponding organoids (Fig. 3n‒q). Consistent with the observations under stress conditions, the corticosterone-induced effect disappeared in the secondary organoids (Supplementary Fig. S5s‒v), suggesting that the deleterious effect of corticosterone on ISCs is also reversible.

Overall, these data indicate that stress activates the HPA axis to release corticosterone, which does not increase in response to stress in ADX mice (Supplementary Fig. S5w), thus relaying its detrimental effect to ISCs and leading to the disruption of intestinal homeostasis.

### Stress-induced corticosterone directly regulates ISCs via NR3C1

**Fig. 4:**
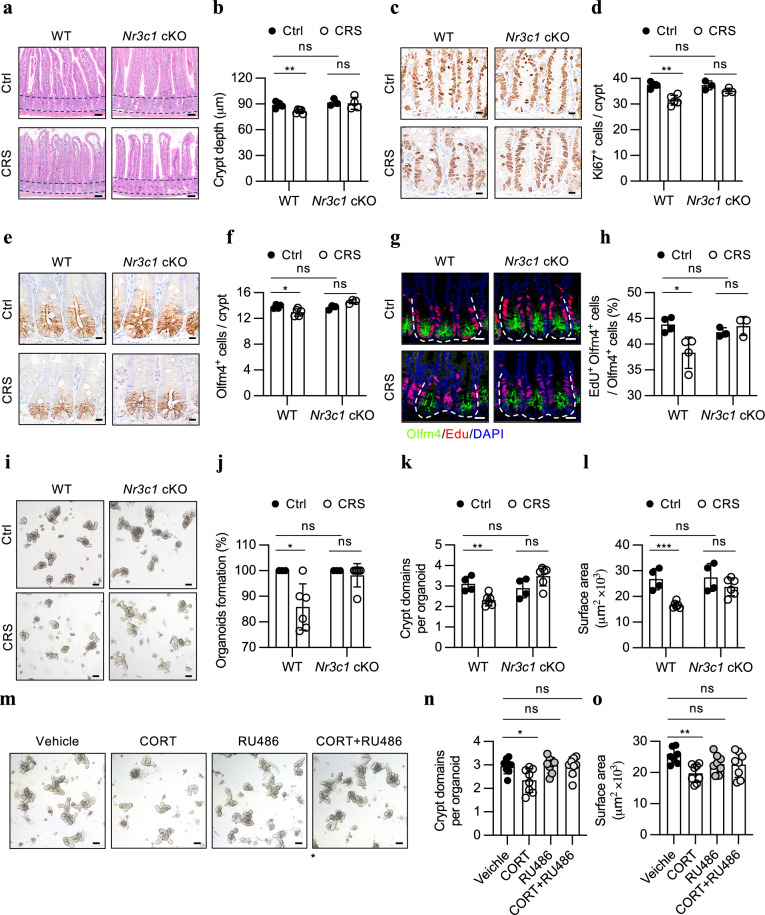
Stress-induced corticosterone impairs ISCs via NR3C1. **a**, **b** H&E staining (**a**) and quantification of crypt depth (**b**) in the jejuna of WT (*Nr3c1*^*fl/fl*^) + Ctrl (*n* = 4), WT + CRS (*n* = 5), *Nr3c1* cKO (*Lgr5;Nr3c1*^*fl/fl*^) + Ctrl (*n* = 3) and *Nr3c1* cKO+CRS (*n* = 4) mice. Scale bar: 50 µm. **c**, **d** Ki-67 staining (**c**) and quantification of Ki-67^+^ cells per crypt (**d**) in the jejuna of the WT + Ctrl (*n* = 4), WT + CRS (*n* = 5), *Nr3c1* cKO + Ctrl (*n* = 3) and *Nr3c1* cKO + CRS (*n* = 3) mice. Scale bar: 10 µm. **e**, **f** Olfm4 staining (**e**) and quantification of Olfm4^+^ cells per crypt (**f**) in the jejuna of the WT + Ctrl (*n* = 4), WT + CRS (*n* = 5), *Nr3c1* cKO + Ctrl (*n* = 3) and *Nr3c1* cKO + CRS (*n* = 3) mice. Scale bar: 10 µm. **g**, **h** EdU and Olfm4 co-staining (**g**) and the proportion of EdU^+^Olfm4^+^ cells per crypt (**h**) in the jejuna of the WT + Ctrl (*n* = 4), WT + CRS (*n* = 4), *Nr3c1* cKO + Ctrl (*n* = 3) and *Nr3c1* cKO + CRS (*n* = 3) mice. EdU (0.2 mg/25 g body weight) was administered to mice 1.5 h prior to sacrifice. Scale bar: 20 µm. **i**‒**l** Representative images of day 4 organoids (**i**) and quantification of organoid formation (**j**), bud number (**k**) and surface area (**l**) derived from the WT + Ctrl (*n* = 4), WT + CRS (*n* = 6), *Nr3c1* cKO + Ctrl (*n* = 4) and *Nr3c1* cKO + CRS (*n* = 6) mice. Scale bar: 100 µm. **m** Representative images of day 4 organoids after ex vivo treatment with 200 μM CORT or 200 μM CORT simultaneously supplemented with 5 μM RU486, an antagonist of the glucocorticoid receptor. Scale bar: 100 µm. Quantification of the bud number (**n**) and surface area (**o**) of the organoids in (**m**) (*n* = 8). The data are presented as the mean ± SD. The statistical analysis was performed by an unpaired two-tailed Student’s *t*-test for normally distributed data or the Mann‒Whitney test for non-normally distributed data. For multiple group comparisons, one-way ANOVA with Dunnett’s multiple comparisons test was used for normally distributed data, and the Kruskal‒Wallis test with Dunn’s multiple comparisons test was used for non-normally distributed data. ns, *p* ≥ 0.05, *p < 0.05, **p < 0.01, ***p < 0.001.

Corticosterone exerts its broad physiological and pathological functions by interacting with the glucocorticoid receptor (GR), also known as NR3C1, a member of the nuclear receptor family of ligand-activated transcription factors^25^. To identify the target cell types of corticosterone, we performed immunofluorescence staining of NR3C1 in the small intestine and observed high expression of this gene in epithelial cells, including ISCs (Supplementary Fig. S6a), suggesting that corticosterone might directly act on ISCs. To assess this hypothesis, we depleted *Nr3c1* in ISCs using the *Lgr5-creERT2* driver in mice and subsequently treated the animals with CRS (Supplementary Fig. S6b‒d). Like in the ADX mice, the *Lgr5-creERT2; NR3C1*^*fl/fl*^ mice also exhibited no reductions in the crypt depth and the Ki-67-positive cell number under stress conditions (Fig. 4a‒d). Moreover, *Nr3c1* depletion in ISCs prevented the stress-induced decrease in their number and proliferative activity (Fig. 4e‒h). Consistent with these findings, the ex vivo organoid culture system revealed that the regenerative capacity of ISCs from the *Lgr5-creERT2; NR3C1*^*fl/fl*^ mice was almost completely restored (Fig. 4i‒l). Therefore, we conclude that stress-elevated corticosterone could directly impair ISC activity by interacting with NR3C1.

To further confirm the direct regulatory effect of corticosterone on ISCs, we supplemented the culture media with corticosterone in the ex vivo intestinal organoid culture system and found that corticosterone significantly suppressed the growth of intestinal organoids in a dose-dependent manner (Supplementary Fig. S6e‒g). To determine whether this inhibitory effect of corticosterone on organoid growth is mediated by NR3C1, we simultaneously added its antagonist RU486 (mifepristone), and observed an effectively restored growth of intestinal organoids (Fig. 4m‒o). Furthermore, immunofluorescence staining for GFP and Ki67 in these organoids showed that corticosterone treatment significantly reduced the number of GFP-positive cells and their proliferative activity, whereas RU486 treatment effectively reversed these effects (Supplementary Fig. S6h‒j).

Taken together, these findings demonstrate that stress-induced corticosterone can act directly on ISCs to impair their activity via NR3C1.

### Corticosterone alters the ISC transcriptome

**Fig. 5:**
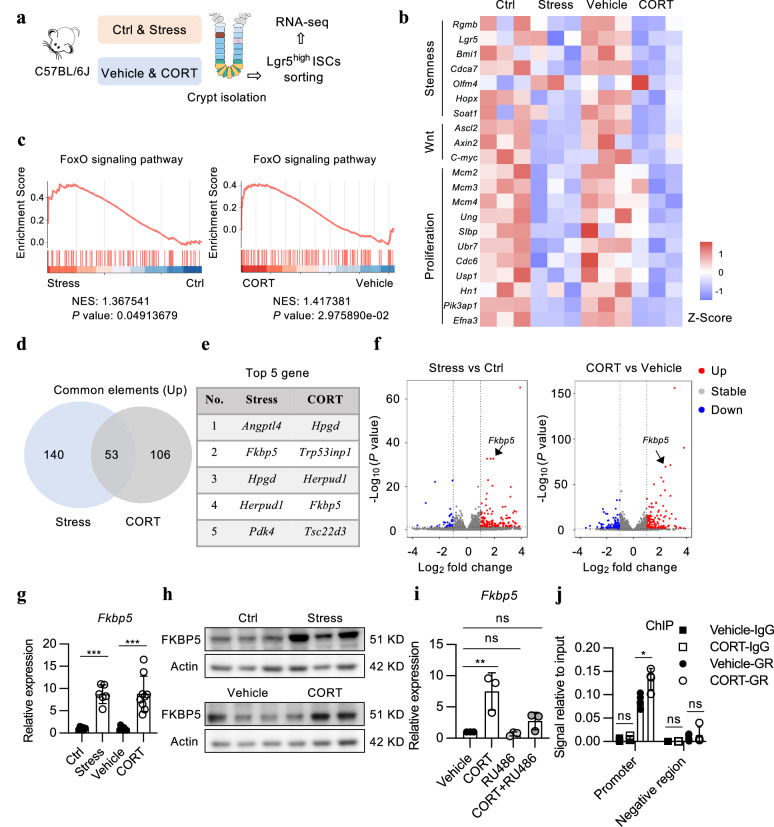
FKBP5 is upregulated by corticosterone in ISCs. **a** Schematic diagram of RNA-seq analysis of ISCs isolated from the small intestine of Ctrl, restraint stress-treated, vehicle-treated and CORT-treated mice. *Lgr5-EGFP-IRES-creERT2* mice were exposed to restraint stress or CORT treatment for 1 day (*n* = 3). The small intestinal crypt fractions were isolated and dissociated into single-cell suspensions. ISCs were sorted by using flow cytometry and subjected to RNA-seq analysis. **b** Heatmap showing the expression of stem cell marker genes, Wnt-related genes and proliferation-related genes in ISCs isolated from the small intestine of Ctrl, restraint stress-treated, vehicle-treated and CORT-treated mice. **c** Gene set enrichment analysis (GSEA) of transcriptome profiles showing high enrichment of FoxO signaling pathway-related genes in ISCs from the stressed (vs. Ctrl) and CORT-treated (vs. vehicle) mice. Differentially expressed gene, > 2-fold; *P*_adj_ value < 0.05. **d** Venn diagram showing overlapping upregulated genes in ISCs from the stress-treated (vs. Ctrl) and CORT-treated (vs. vehicle) mice. **e** Top 5 upregulated genes in ISCs from the stress-treated (vs. Ctrl) and CORT-treated (vs. vehicle) mice. **f** Volcano plots showing the genes differentially expressed in ISCs from the stress-treated (vs. Ctrl) and CORT-treated (vs. vehicle) mice. **g** RT–qPCR analysis of *Fkbp5* expression in the small intestinal crypts from the 1-day restraint stress- and CORT-treated mice. *n* = 6 and 9 mice for the restraint stressed and CORT-treated groups, respectively. **h** Western blotting for FKBP5 in the small intestinal crypts from the 2-day stress- and CORT-treated mice. β-actin was used as a loading control. **i** RT–qPCR analysis of *Fkbp5* expression in intestinal organoids after ex vivo treatment with 200 μM CORT or 200 μM CORT simultaneously supplemented with 5 μM RU486 (*n* = 3). **j** A chromatin immunoprecipitation assay was performed using an antibody against NR3C1 on crypts isolated from the WT mice treated with vehicle or CORT for 1 day (*n* = 4). IgG was used as a negative control, and the enrichment of NR3C1 binding to the *Fkbp5* promoter was quantified using qPCR. The data are presented as the mean ± SD. The statistical analysis was performed by unpaired two-tailed Student’s *t*-test for normally dis*t*ributed data or the Mann‒Whitney test for non**-**normally distributed data and by one-way ANOVA with Dunnett’s multiple comparisons test or the Kruskal**‒**Wallis test with Dunn’s multiple comparisons test for non-normally distributed data. ns, *p* ≥ 0.05, *p < 0.05, **p < 0.01, ***p < 0.001.

To determine the molecular mechanisms driving the deleterious effect of stress on ISCs via corticosterone, we conducted RNA-seq in purified Lgr5-EGFP^hi^ ISCs from the mice subjected to a single exposure to either restraint stress or corticosterone injection (Fig. 5a; Supplementary Fig. S7a) at a stage when no obvious intestinal phenotypic differences were apparent, as suggested by Zhang et al.^13^. Analysis of the expression of signature genes for different types of intestinal epithelial cells confirmed that we successfully enriched ISCs (Supplementary Fig. S7b). Further analysis revealed that both restraint stress and corticosterone treatment dramatically suppressed the expression of stem cell signature genes and classical Wnt target genes as well as various genes known to be involved in the regulation of proliferative activity (Fig. 5b)^26,27^.

Pairwise comparisons between samples [stress vs. control (Ctrl); corticosterone vs. vehicle] were then performed to identify differentially expressed genes (2-fold change, *P*_adj_ < 0.05). Kyoto Encyclopedia of Genes and Genomes (KEGG) pathway enrichment analysis revealed that the forkhead box O (FoxO) signaling pathway, adipocytokine signaling pathway, and circadian rhythm were enriched in both the restraint stress- and corticosterone-treated groups (Supplementary Fig. S7c‒f). Gene set enrichment analysis (GSEA) further revealed that FoxO and adipocytokine signaling pathway components were significantly upregulated in these two groups (Fig. 5c; Supplementary Fig. S7g, h).

### Corticosterone impairs ISCs through the FKBP5-AKT-FoxO signaling pathway

The FoxO signaling pathway is involved in various physiological processes, including cell cycle regulation^28^. Indeed, RT‒qPCR confirmed that corticosterone or stress treatment significantly increased the expression of signature genes in the FoxO signaling pathway within the crypt (Supplementary Fig. S8a, b).

To explore how stress or corticosterone treatment activates the FoxO signaling pathway, we examined the genes that were commonly differentially expressed between the stress- and corticosterone-treated groups, and identified 53 genes with increased expression and 11 genes with decreased expression (Fig. 5d; Supplementary Fig. S7i). Among these candidates, *Fkbp5* was significantly upregulated (Fig. 5e, f), which has been reported to play an important role in regulating the FoxO signaling pathway^29,30^. Therefore, we performed independent experiments using different biological sample sets and confirmed the upregulation of *Fkbp5* in the crypts of small intestine from the mice subjected to stress exposure or corticosterone treatment (Fig. 5g, h). Similarly, the upregulation of FKBP5 induced by stress and corticosterone was also confirmed in the crypts isolated from the colon (Supplementary Fig. S8c, d). Furthermore, we demonstrated that corticosterone enhanced *Fkbp5* transcription through interacting with NR3C1 in the ex vivo organoid system (Fig. 5i). Meanwhile, we conducted a chromatin immunoprecipitation (ChIP) assay and validated that corticosterone facilitated the binding of NR3C1 to the *Fkbp5* promoter (Fig. 5j).

**Fig. 6:**
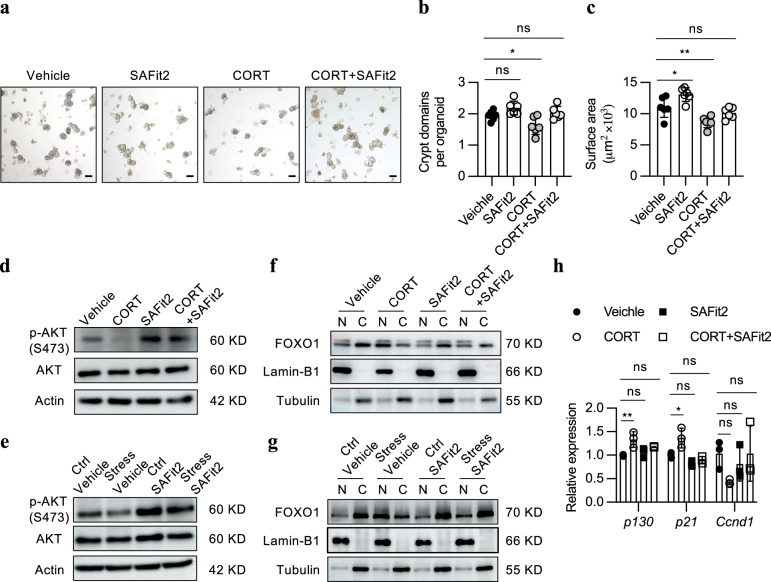
FKBP5-AKT signaling is responsible for the effect of corticosterone on ISCs. **a** Representative images of day 4 organoids after ex vivo treatment with 200 μM CORT, 0.25 μM SAFit2, or 200 μM CORT supplemented with 0.25 μM SAFit2. SAFit2, a highly selective FKBP5 inhibitor. Scale bar: 100 µm. **b**, **c** Quantification of the bud number (**b**) and surface area (**c**) of the organoids in **a** (*n* = 6). **d**, **e** Western blotting for AKT and pAKT (S473) in the small intestinal crypts of the vehicle-treated, CORT-treated (5 mg/kg/day), SAFit2-treated (20 mg/kg/day) and CORT + SAFit2-treated mice (**d**) and in the small intestinal crypts of the Ctrl + vehicle-treated, stress+vehicle-treated, Ctrl + SAFit2-treated and stress+SAFit2-treated mice (**e**). Mice were exposed to those treatments for 2 days. β-actin was used as a loading control. **f**, **g** Western blotting for FOXO1 in nuclear and cytoplasmic proteins isolated from the small intestinal crypts of the vehicle-treated, CORT-treated, SAFit2-treated and CORT + SAFit2-treated mice (**f**) and from the small intestinal crypts of the Ctrl + vehicle-treated, stress + vehicle-treated, Ctrl + SAFit2-treated and stress+SAFit2-treated mice (**g**). Mice were exposed to those treatments for 2 days. Lamin B1 and tubulin were used as nuclear proteins and cytoplasmic protein loading controls, respectively. **h** RT–qPCR analysis of *p130, p21*, and *Ccnd1* expression in small intestinal crypts from the 1-day vehicle-treated, CORT-treated, SAFit2-treated and CORT + SAFit2-treated mice (*n* = 3). The data are presented as the mean ± SD. The statistical analysis was performed by one-way ANOVA with Dunnett’s multiple comparisons test. ns, *p* ≥ 0.05, *p < 0.05, **p < 0.01.

To determine whether FKBP5 mediates the deleterious effect of corticosterone on ISCs, we supplemented SAFit2, a highly selective FKBP5 inhibitor, along with corticosterone in the ex vivo organoid system, and found that the inhibited growth of intestinal organoids was almost recovered (Fig. 6a‒c). FKBP5 has been reported to form a complex with AKT and PHLPP through its FKBP domain and tetratricopeptide repeat (TRP) domain, respectively, thus facilitating PHLPP-mediated dephosphorylation of AKT at Ser473 and negatively regulating AKT activity^29,31^. This process subsequently leads to the dephosphorylation of FOXO1 and promotes its nuclear translocation^32^. Consistent with these findings, western blotting revealed that corticosterone or stress treatment resulted in a dramatic decrease in the level of p-AKT (S473) in crypts isolated from both the small intestine and the colon (Supplementary Fig. S8e‒h). However, SAFit2 treatment significantly restored the level of p-AKT (S473) and reduced the nuclear translocation of FOXO1 (Fig. 6d‒g), thus ultimately reducing the expression of cell cycle inhibitors, namely *p130* and *p21* (Fig. 6h).

Taken together, these data indicate that stress-induced elevation of corticosterone activates NR3C1 to promote *Fkbp5* transcription and inhibit Akt phosphorylation. The inactivity of AKT subsequently leads to FOXO1 nuclear translocation, which suppresses the proliferative activity of ISCs.

### ISC dysfunction contributes to the stress-driven exacerbation of colitis

Many epidemiological studies have consistently reported an association between psychological stress and IBD^33–35^. Using the dextran sodium sulfate (DSS)-induced colitis model, it has been demonstrated that chronic stress exacerbates colitis severity in mice through mechanisms involving alterations in the gut microbiota and the induction of inflammatory enteric glia^7,20^. Considering the critical role of ISC in epithelial regeneration during intestinal inflammation, we hypothesized that ISC dysfunction leads to the impairment of epithelial repair, which might be one of the key factors contributing to the stress-exacerbated colitis.

To confirm the aggravative effect of chronic stress on intestinal inflammation, we employed the DSS-induced colitis model (Supplementary Fig. S9a). In line with previous reports, chronic stress significantly exacerbated DSS-induced colitis, as evidenced by greater weight loss, a higher disease activity index (DAI) and shorter colon length (Supplementary Fig. S9b‒e). Consistent with these findings, chronic stress also led to aggravated colonic histopathology, characterized by extensive epithelial damage, inflammatory infiltrates, and increased expression of pro-inflammatory cytokines in the mucosal tissue (Supplementary Fig. S9f‒i). Importantly, along with the severe epithelial damage, chronic stress caused a dramatic reduction in both the number of Lgr5-positive cells and the Ki-67-positive frequency in these cells (Supplementary Fig. S9j‒l), suggesting that ISC dysfunction may impair the epithelial repair during inflammation, and contribute to the stress-enhanced susceptibility to colitis.

**Fig. 7:**
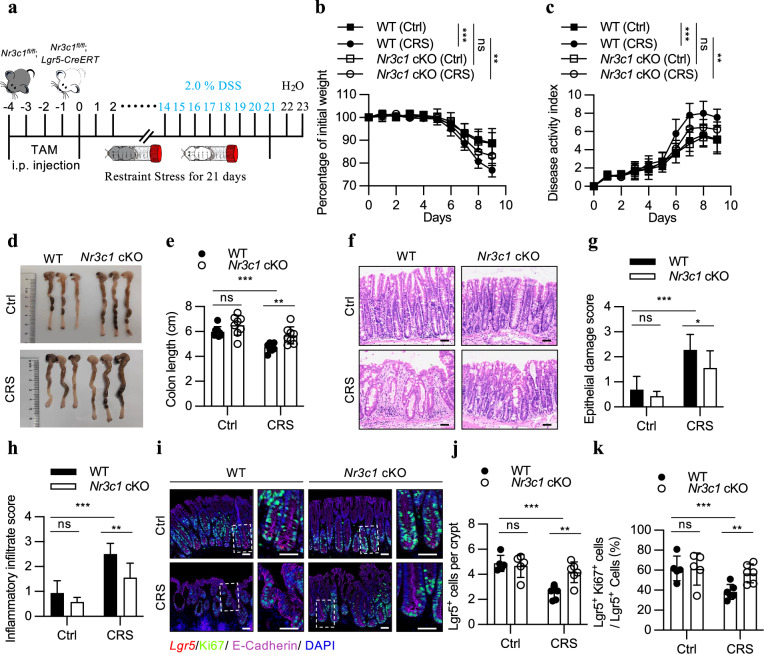
*Nr3c1* deficiency in ISC mitigates the stress-exacerbated colitis. **a** Schematic of the dextran sulfate sodium (DSS)-induced colitis model. WT and *Nr3c1* cKO mice received a 5-day tamoxifen (TAM, 2 mg/25 g body weight) injection. A subset of the mice was were subjected to CRS treatment. During the last week of the three-week CRS treatment, mice were given 2.0% DSS in drinking water for seven days, followed by a 2-day recovery period with normal drinking water. **b**, **c** Body weight (**b**) and disease activity index (DAI) (**c**) of WT (*Nr3c1*^*fl/fl*^) + Ctrl (*n* = 8), WT + CRS (*n* = 9), *Nr3c1* cKO (*Lgr5;Nr3c1*^*fl/fl*^) + Ctrl (*n* = 8) and *Nr3c1* cKO + CRS (*n* = 9) mice during 2.0% DSS treatment. Body weight was monitored daily and expressed as a percentage of the initial body weight. **d**, **e** Representative image of colon (**d**) and quantification of colon length (**e**) of WT + Ctrl, WT + CRS, *Nr3c1* cKO + Ctrl, *Nr3c1* cKO + CRS mice (*n* = 8) after 7-day DSS treatment and 2-day recovery period. **f**‒**h** Representative H&E staining image (**f**), epithelial damage score (**g**), and inflammatory infiltrate score (**h**) of colonic section from WT + Ctrl (*n* = 8), WT + CRS (*n* = 9), *Nr3c1* cKO + Ctrl (*n* = 7), *Nr3c1* cKO + CRS (*n* = 9) mice after 7 days of DSS treatment and a 2-day recovery period. Scale bar: 50 µm. **i** Representative image of in situ hybridization for Lgr5 combined with immunofluorescence staining for Ki67 and the epithelial marker cadherin (E-cadherin) in the colons of Ctrl and CRS-treated mice with 2.0% DSS treatment. Scale bar: 50 µm. **j**, **k** Quantification of percentage of Lgr5^+^ cells per crypt (**j**), and the proportion of Lgr5^+^ cells expressing Ki-67 (**k**) in the colons of WT + Ctrl (*n* = 5), WT + CRS (*n* = 6), *Nr3c1* cKO + Ctrl (*n* = 5) and *Nr3c1* cKO + CRS (*n* = 6). The data are presented as the mean ± SD. The statistical analysis was performed by an unpaired two-tailed Student’s *t*-test for normally distributed data or the Mann‒Whitney test for non-normally distributed data. ns, *p* ≥ 0.05, *p < 0.05, **p < 0.01, ***p < 0.001.

To test this hypothesis, we induced colitis in CRS-treated *Lgr5-creERT2; NR3C1*^*fl/fl*^ mice. Western blotting revealed that *Nr3c1* deletion significantly restored the level of p-AKT (S473) and reduced the nuclear translocation of FOXO1 in the crypts of stress-treated mice (Supplementary Fig. S10a, b). This restoration prevented the stress-induced decrease in ISC number and proliferative activity (Fig. 4e‒h). Building on this, we analyzed the impact of this restored ISC function by *Nr3c1* deletion on the stress-aggravated colitis (Fig. 7a). The results showed that while ISC-specific *Nr3c1* deletion itself did not affect the severity of DSS-induced colitis, it significantly alleviated the aggravation of colitis severity caused by CRS. Specifically, compared to CRS-treated wild-type mice, ISC-specific *Nr3c1* deletion mice exhibited partial recovery in body weight, a reduced DAI, and restored colon length (Fig. 7b‒e). These improvements were accompanied by a reduction in epithelial damage and inflammatory infiltrates (Fig. 7f‒h). Furthermore, the decline in Lgr5-positive cell numbers and their proliferative capacity in CRS-treated WT mice was substantially reversed in these mice (Fig. 7i‒k).

Collectively, although not a complete restoration, ISC-specific *Nr3c1* deletion significantly protected mice from the stress-driven exacerbation of DSS-induced colitis, suggesting that ISC dysfunction is one of major contributors to the worsening of colitis under chronic stress conditions.

## Discussion

Chronic psychological stress profoundly affects the gastrointestinal tract and is strongly associated with the occurrence and development of gastrointestinal disorders^6^. In this study, we identified an intrinsic brain-to-gut pathway that relays the detrimental effect of psychological stress to ISCs and revealed its contribution to the stress-increased susceptibility to colitis. Our findings not only provide new insights into cellular and molecular mechanism through which psychological stress affects intestinal homeostasis but also identify a critical regulator of ISCs at the systemic level, highlighting the importance of mental well-being for gut health.

ISCs are renowned for their remarkable proliferative capacity, which enables them to divide and generate new cells to replenish damaged or lost cells in the intestinal lining^10^. Any disturbance that compromises the activity and function of ISCs would sensitize the intestinal epithelium to other environmental stimuli, potentially laying the foundation for gastrointestinal disorders^11^. In the human population, it has been reported that individuals with a history of mental disorders are more likely to be diagnosed with IBD^36^. Consistently, our study and others clearly demonstrate that chronic stress can facilitate the development of DSS-induced colitis in mice (Supplementary Fig. S9)^7,20^. Importantly, restoration of ISC function by *Nr3c1* deletion significantly mitigates this aggravated effect (Fig. 7), suggesting a critical role for ISC dysfunction in the stress-increased susceptibility to developing colitis. It should be noted that *Nr3c1* deletion only partially ameliorates DSS-induced colitis, indicating the existence of other mechanisms connecting stress to intestinal inflammation. Indeed, it has been reported that chronic stress can also promote intestinal inflammation by disturbing the gut microbiota or inducing inflammatory enteric glia in colonic mucosa^7,20^. Taken together, our study underscores the paramount importance of managing mental health for each individual to lower the risk of impeding intestinal homeostasis and developing intestinal inflammation stemming from ISC dysfunction.

The brain-to-gut regulatory pathway involves intricate neural and hormonal signaling that enables brain to influence the activities of various intestinal effector cells, including epithelial cells, enteric neurons, interstitial cells of Cajal and enterochromaffin cells^2^. Individuals experiencing psychological stress exhibit disturbances in this regulatory process, increasing their susceptibility to gut disorders. For instance, a recent study found that psychological stress and chronically elevated glucocorticoid levels can induce inflammatory enteric glia, exacerbating IBD flare^7^, underscoring the pivotal role of neuroendocrine mediators in relaying the adverse effects of psychological stress to the gut. Additionally, the gut microbiome, which can be dynamically shaped by stress, is widely recognized as a key regulator of brain‒gut crosstalk^6^. Recently, Wei et al. revealed that stress-induced microbial dysbiosis, mediated by sympathetic activation, triggers defects in ISC commitment to secretory cell lineage, emphasizing the significance of this external factor for ISC function, and prompting questions about whether the brain can communicate with ISCs via intrinsic organismal responses and which responses contribute to transmitting stress influences to ISC behaviors^18^. Our study bridges this gap by showing that the elevation of systemic corticosterone levels, resulting from the activation of the HPA axis during stress, directly impairs ISC proliferation and functionality via its receptor NR3Cl, enriching our understanding of the intrinsic brain-to-ISC regulatory pathway.

Notably, although we employed a CRS model similar to that used by Wei et al., we observed distinct effects on ISCs, particularly regarding their differentiation into secretory cells. Wei et al. reported that stress disturbs mitochondrial respiration of intestinal stem cells and contributes to a transferrable impairment of secretory cell differentiation in mice through the gut commensal *Lactobacillus murinus* enrichment and subsequent increases in the production of indole-3-acetate (IAA). We hypothesize that the gut microbiota may play a critical role in these differences. In our experimental setting, we did not observe significant changes in either *L. murinus* or IAA levels following CRS (data not shown). It is well-known that the composition of the gut microbiota is influenced by a variety of factors, including genetic background, environmental conditions, and animal housing facilities^37–39^. Variations in these factors may have resulted in differences in the gut microbiota composition following CRS treatment, potentially explaining the divergent findings between our study and that of Wei et al.

Corticosterone has been reported to suppress the expression of *Gas6* in the dermal papillae and govern hair follicle stem cells (HFSCs) quiescence, indicating its indirect regulatory role in stem cell. Here, we found that this stress hormone can act directly on ISCs through its receptor NR3C1, suggesting diverse pathways and distinct mechanisms involved. In addition, physiological stress can also affect somatic stem cells through catecholamines, such as noradrenaline (also known as norepinephrine). It has been reported that chronic stress can activate sympathetic nerves to release this catecholamine, thus driving melanocyte stem cells (MSCs) proliferation by directly binding to the β_2_-adrenergic receptor, or promoting hematopoietic stem cells (HSCs) expansion by modulating bone marrow niche cells through the β_3_-adrenergic receptor^12,13^. In our study, we observed an increase trend of this hormone in the CRS-exposed mice, but we did not observe any influence on ISC function. Collectively, our finding provides an example in which somatic stem cells are directly regulated by a systemic factor from the adrenal glands, contributing to our current understanding of the regulatory mechanisms that relay the effect of stress to somatic stem cells.

Glucocorticoids, known as cortisol in humans and corticosterone in rodents, are generally recognized as anti-inflammatory agents^40^. Clinically, glucocorticoid receptor agonists such as prednisone and dexamethasone have been widely used to treat inflammatory diseases, including IBD^41^. However, data revealed that only short-term treatment yields beneficial outcomes in patients with IBD^42,43^. Recent research has helped elucidate this phenomenon. Chronically elevated glucocorticoid levels can induce inflammatory enteric glia, which subsequently mediate the appearance and accumulation of TNF-producing monocytes, further exacerbating colitis^7^. In addition to aggravating IBD flares, corticosteroid administration has also been identified as a negative predictor of mucosal healing, a crucial therapeutic goal in IBD, but the precise underlying mechanisms are not yet fully understood^44^. Since mucosal repair primarily depends on the orchestrated regeneration of IECs driven by ISCs, our findings of a significant reduction in both the number and activity of ISCs in the presence of elevated corticosterone levels provide a plausible explanation for the potential mechanisms underlying this clinical phenomenon. In the future, it will be intriguing to investigate whether the mechanism that we have uncovered here contributes to impaired mucosal healing using both the mouse models and clinical IBD samples.

Some limitations of the study should also be acknowledged. Our study predominantly utilized mouse models and an ex vivo organoid system to explore the impact of psychological stress on ISCs and to uncover the underlying cellular and molecular mechanisms. Nonetheless, while these models provide valuable insights, they may not replicate the intricacies of the human intestine. Substantial disparities in genetics, systemic responses to stressors, and the intestinal microenvironment exist between mice and humans, and these differences could affect the influence of psychological stress on ISCs and homeostasis.

## Materials and methods

### Mouse breeding, maintenance and treatment

*Nr3c1*^*fl/fl*^ mice were generated by GemPharmatech. *Lgr5*^*EGFP-IRES-CreERT2*^ mice (Jackson Laboratory; stock number: 008875) and C57BL/6 J mice were obtained from Jackson Laboratory and Shanghai Slack Company, respectively. For the *Nr3c1* knockout experiments, both control mice (*Nr3c1*^*fl/fl*^) and *Lgr5*^*EGFP-IRES-CreERT2*^; *Nr3c1*^*fl/fl*^ mice received an equal dose of tamoxifen (Sigma‒Aldrich, T5648) dissolved in corn oil. In brief, mice were intraperitoneally injected with tamoxifen daily for 5 consecutive days (2 mg/25 g body weight). For analysis of proliferating cells, EdU (Thermo Fisher, A10044) was dissolved in sterile saline and injected intraperitoneally into mice (0.2 mg/25 g body weight) 1.5 h before sacrifice. Mice were assigned to experimental groups based on their genotype (Supplementary Table S1) and randomized into different treatment groups. All mice were maintained under pathogen-free conditions at the Laboratory Animal Centre of Zhejiang University, and provided with water and a standard laboratory diet ad libitum, except where otherwise noted. Both male and female age-matched mice between 8‒10 weeks of age were utilized for the experiments. Alternate between data or samples collection from mice subjected to different treatments was adopted to minimize potential confounders. All animal studies were performed in compliance with the guide for the care and use of laboratory animals, adopting the protocol approved by the Medical Experimental Animal Care Commission of ZJU (#ZJU20220175).

### Stress procedures

CRS was conducted following a previously described method^20^. Briefly, mice were subjected to 3 h of confinement per day at random times in the morning or afternoon over a 3-week period using well-ventilated 50 mL centrifuge tube. During restraint, the CRS mice were unable to move freely within the tube. To control for the potential effects of food and water deprivation during the restraint periods, the control mice were similarly deprived of food and water for the same duration as the CRS mice without the physical restraint.

For CUS, a modified version of established protocols was used^12^. Briefly, mice were exposed to various stressors over a 2-week period. Each day, two of the following stressors were randomly applied: cage tilt for 2 h, isolation for 3.5 h, restraint for 3 h, water stress for 2 h, damp bedding for 2 h, sawdust changes 4 times within a 2-h span, and an empty cage for 4 h.

### ADX

ADX was performed in accordance with previously established protocols^14^. C57BL/6 J mice were anesthetized, and precise incisions were made directly above each adrenal gland on the dorsal skin. In mice undergoing ADX, both adrenal glands were surgically removed. Sham-operated mice underwent identical procedures, except that their adrenal glands were left intact. Since adrenal glands are involved in the secretion of aldosterone to regulate the water-salt balance, both ADX and sham mice were provided with drinking water supplemented with 1% w/v saline solution. One week after recovery from surgery, the mice were subjected to the restraint stress for a duration of 3 weeks. The efficacy of the surgical procedure was evaluated by measuring corticosterone levels in both ADX and sham mice.

### FST

The FST was performed according to a previously described procedure^45^. Briefly, both the control and stress-treated mice were gently placed individually into a transparent cylinder filled with water (25 °C) to a depth where the mice could not reach the bottom with their hind paws for 6 min. The entire process was recorded, and the immobility time was measured during the last 4 min. Mice were considered immobile if they ceased struggling, except for the necessary movement to keep the nose above the water.

### Colitis induction with DSS

Mice were administrated with DSS through drinking water to induce acute colitis as previously described^46^. In brief, sex-matched 8- to 10-week-old mice were subjected to restraint stress for 21 days. On day 14 of this period, both stressed and control mice were fed with DSS (2.0% w/v; 36-50 kDa; MP biomedicals) in drinking water for 7 days to induce colitis. Mice were sacrificed, and intestinal tissues were collected either at the end of colitis induction or two days after returning to regular drinking water. Weight, stool consistency and bleeding score were recorded daily until the end of the experiment. The length of the entire colon was measured, and colonic tissue was collected for subsequent histological and molecular analyses.

### DAI

The clinical sigh of mouse colitis was assessed based on the DAI score, which includes body weight loss, occult blood, stool consistency, as previously described^46^. Mice were scored blindly during the colitis induction. In brief, weight loss score was determined as follows: 0, no weight loss; 1, loss of 1%–5% original weight; 2, loss of 6%–10% original weight; 3, loss of 11%–15% original weight; and 4, loss of 15%–20% original weight. Stool score was determined as follows: 0, well-formed pellets; 1, semi-formed stools that did not adhere to the anus; 2, pasty semi-formed stool that adhered to the anus; and 3-4, liquid stools that adhered to the anus. Bleeding score was determined as follows: 0, no blood by using Hemoccult (Beckman Coulter) analysis; 1, positive Hemoccult; 2, visible blood traces in stool; and 3-4, gross rectal bleeding.

### Histological score

The proximal and distal colonic tissues were fixed in 4% paraformaldehyde for 24 h at room temperature. Following paraffin embedding, 3-µm thick sections were prepared and stained with H&E. Histological assessment of colitis was performed blindly by board-certified pathologist based on the criteria that consist of epithelial damage and inflammatory infiltrate in the mucosa, submucosa, and muscularis/serosa. In brief, the epithelial damage score was determined as follows: 0, absent; 1, the damage of discontinuity; 2, the damage of mucosa erosion; 3, large areas of ulceration. Inflammatory infiltrate score was determined as follows: 0, absent; 1, slight; 2, present; 3, heavy.

### Measurement of stress hormones

For CORT treatment, CORT (Med Chem Express, HY-B1618) was dissolved in 20% sulfobutylether-β-cyclodextrin and intraperitoneally injected either once or daily for 3 weeks at a dose of 5 mg/kg. Plasma CORT levels were determined using an ELISA kit (ARBOR ASSAYS, K014-H1) following the manufacturer’s instructions. Plasma adrenaline and NE were quantified utilizing LC–MS/MS, and all samples were analyzed using a Waters ACQUITY UPLC I-Class coupled with a Waters Xevo TQ-XS tandem quadrupole mass spectrometer.

### Drug treatment

For CORT treatment, CORT (Med Chem Express, HY-B1618) was dissolved in 20% sulfobutylether-β-cyclodextrin and intraperitoneally injected either once or daily for 3 weeks at a dose of 5 mg/kg. The control mice received vehicle (20% sulfobutylether-β-cyclodextrin). For NE treatment, NE (MedChemExpress, HY-13715) was dissolved in 20% sulfobutylether-β-cyclodextrin and intraperitoneally injected daily for 3 weeks at a dose of 1 mg/kg. For SAFit2 treatment, SAFit2 (MedChemExpress, HY-102080) was dissolved in vehicle containing 10% DMSO, 40% PEG300, 5% Tween-80, and 45% saline (with the solvents added sequentially) and administered intraperitoneally once.

### Histology, immunohistochemistry, immunofluorescence and in situ hybridization assays

Mouse small intestinal samples were fixed in 4% paraformaldehyde for 24 h at room temperature and subsequently embedded in paraffin. For histological staining, 3-µm thick sections were prepared and stained with H&E. PAS (Leagene, DG0005), Alpi (Beyotime, P0321S) and EdU (Beyotime, C0078S) stainings were performed following the manufacturer’s instructions.

For immunohistochemistry, sections were dewaxed, rehydrated and subjected to antigen retrieval using 0.01 M citrate buffer (pH=6) at 95 °C for 20 min. After cooling to room temperature, the slices were incubated with 3% H_2_O_2_ for 10 min to quench endogenous peroxidase activity. Subsequently, the sections were blocked with blocking buffer (Beyotime, P0102) at room temperature for 1 h and then incubated with the primary antibody overnight at 4 °C. A mouse/rabbit streptavidin-biotin system (ZSGB-BIO, SP-9001/9002) was used for the secondary antibody, and hematoxylin was used as a counterstain.

For immunofluorescence, sections were incubated with fluorescence-conjugated secondary antibodies (Invitrogen, A21206/A31572/A31573/A31570/A21202/A31571) and counterstained with DAPI (Thermo, 62248) before being covered with anti-quenching reagent (Beyotime, P0126). The primary antibodies used were against Ki-67 (BD Biosciences, 556003, 1:500), β-catenin (Sigma, C7082, 1:400), lysozyme C (Abcam, ab108508, 1:500), ChgA (Abcam, ab254322, 1:10000), Olfm4 (Cell Signaling Technology, 39141, 1:800), and GFP (Abcam, ab13970, 1:800).

In situ hybridization was performed according to the manufacturer’s instructions (PinpoRNA, PIF1000) using *Lgr5* Probe (PinpoRNA, 141602-B2). After incubation with fluorogenic substrate, the sections were blocked using blocking buffer and subsequently processed for protein detection as described above.

### Intestinal epithelial cell isolation and FACS

A single-cell suspension of intestinal epithelium was obtained following a previously established protocol with minor modifications^47^. The small intestine was cut open longitudinally, and the villi were gently scraped off. Subsequently, the remaining tissue was incubated with 5 mM EDTA in PBS for 10 min at 4 °C. After thorough vortexing, the crypt fractions were collected and filtered through a 70 µm cell strainer (BD Biosciences). The collected crypts were then centrifuged at 80× *g* for 5 min and digested with TrypLE (Gibco, 12604013) at 37 °C for 2 min. The resulting single-cell suspension was further passed through a 40 µm cell strainer (BD Biosciences) and subsequently stained with DAPI (BD Biosciences, 564907), Fixable Viability Dye (eBioscience, 65-0863-14), CD45 (BioLegend, 103105), CD31 (BioLegend, 102407), TER119 (BioLegend, 116207), CD24 (BD Biosciences, 562360), and EpCAM (eBioscience, 17-5791-80). Flow cytometric analysis and FACS were conducted using a BD LSRFortessa and BD FACSAria II, respectively. ISCs were identified as FVD450^–^CD31^–^CD45^–^TER119^–^EpCAM^+^CD24^–^eGFP^hi^. EGFP^low^ progenitors were identified as FVD450^–^CD31^–^CD45^–^TER119^–^EpCAM^+^CD24^–^eGFP^low^.

### Intestinal organoid culture

Following the isolation of intestinal crypts as described above, intestinal organoid culture was conducted. Briefly, the collected crypts were quantified and then resuspended in a mixture of culture media (STEMCELL Technologies, 06005) and Matrigel (Corning, 356231) at a 1:1 ratio, with a density of 200 crypts/50 µL. The cell suspension was plated at the center of the well in a 24-well plate, which was subsequently incubated at 37 °C for 30 min to allow the Matrigel to solidify. Then, 500 µL of culture medium (STEMCELL Technologies, 06005) was added to each well, and the plate was subsequently incubated at 37 °C and 5% CO_2_ for further analysis.

For CORT treatment, ENR medium containing 100 ng/mL EGF (PeproTech, 315-09), 100 ng/mL Noggin (PeproTech, 120-10c), and 500 ng/mL R-spondin 1 (PeproTech, 96-120-38) was used for intestinal organoid culture, and the cells were supplemented with CORT at concentrations of 50, 100 or 200 μM or with vehicle (DMSO) 1 day after seeding. For RU486 treatment, ENR medium was supplemented with RU486 (MedChemExpress, HY-13683) at a concentration of 5 μM. For SAFit2 treatment, the ENR medium was supplemented with SAFit2 at a concentration of 0.25 μM.

### Chromatin immunoprecipitation‒quantitative PCR (ChIP‒qPCR)

A ChIP‒qPCR assay was performed using a SimpleChIP Plus Enzymatic Chromatin IP Kit (Magnetic Beads) (Cell Signaling Technology, 9005) according to the manufacturer’s instructions. The ChIP antibodies used included anti-IgG (Cell Signaling Technology, 2729) and anti-NR3C1 (Cell Signaling Technology, 12041S). The ChIP DNAs obtained from ChIP were used as templates in subsequent qPCRs. The qPCR primers used were designed to encompass the promoter regions of specific genes, and the sequences used are listed in Supplementary Table S2.

### Cellular fractionation

After isolation of the intestinal crypts as described above, the collected crypts were washed twice with cold PBS. Subsequently, the cytoplasmic and nuclear fractions of the crypts were separated using a Nuclear and Cytoplasmic Protein Extraction Kit (Beyotime, P0028) following the manufacturer’s recommended procedure. Protein concentrations were determined using an Enhanced BCA protein assay Kit (Beyotime, P0009), and the obtained samples were then subjected to immunoblotting analysis.

### Immunoblotting

Proteins were extracted utilizing RIPA buffer (Beyotime, P0013B) supplemented with PMSF (Thermo, 36978) and phosphatase inhibitors (Thermo, A32961) in accordance with the manufacturer’s instruction. Protein concentrations were determined using an Enhanced BCA Protein Assay Kit (Beyotime, P0009) and then adjusted to a final concentration of 2 µg/µL. Subsequently, the proteins were separated on an 8% SDS‒PAGE gel (Yeasen, 20324ES62) and transferred onto a PVDF membrane (Millipore, IPVH00010). After blocking with 5% bovine serum albumin (BSA; Sangon Biotech, A600903) diluted in TBS-Tween (0.5%) for 1 h at room temperature, the membrane was incubated with primary antibodies, followed by binding with a horseradish peroxidase (HRP)-conjugated secondary antibody. Signal visualization was achieved using an enhanced chemiluminescence (ECL) substrate (Yeasen, 36208ES76), and images were acquired with a GE Amersham Imager 680. The primary antibodies used in this study included rabbit monoclonal anti-GR (NR3C1) (Cell Signaling Technology, 12041S), mouse monoclonal anti-FKBP5 (Cell Signaling Technology, 12210S), rabbit polyclonal anti-Akt (Cell Signaling Technology, 9272S), rabbit monoclonal anti-phospho-Akt (Ser473) (Cell Signaling Technology, 4060S), rabbit polyclonal anti-FOXO1 (Proteintech, 18592-1-AP), mouse monoclonal anti-beta actin (Proteintech, HRP-66009), mouse monoclonal anti-alpha tubulin (Proteintech, HRP-66031) and rabbit polyclonal anti-lamin B1(Proteintech, 12987-1-AP).

### RNA isolation

For all RNA manipulations, the equipment was sterilized according to a standard laboratory protocol, and diethylpyrocarbonate-treated water was utilized. Total RNA was extracted for mRNA analysis using TRIzol reagent according to the manufacturer’s instruction. The quality and quantity of the RNA were measured with a Nanodrop 2000 spectrophotometer.

### RT‒qPCR

RNA was extracted from intestinal crypts or organoids, cell lines, and mouse tissues using TRIzol Reagent (TaKaRa, 9109) according to the manufacturer’s instruction. Subsequently, cDNA libraries were synthesized using the PrimeScript™ RT reagent Kit (TaKaRa, RR037B). qPCR was performed using TB Green^®^ Premix Ex Taq™ (TaKaRa, RR420B) on a LightCycler^®^ 480 instrument (Roche). Ct values were normalized with *β-actin* (*Actb*) as the internal control. The primers used are listed in Supplementary Table S3.

### RNA sequencing and analysis

Total RNA was used as the input material for RNA sample preparation. Fragmentation was carried out using divalent cations under elevated temperature in First Strand Synthesis Reaction Buffer (5×). First-strand cDNA was synthesized using a random hexamer primer and M-MLV reverse transcriptase, with RNase H used to degrade the RNA. Subsequently, second-strand cDNA synthesis was performed using DNA polymerase I and dNTPs. After amplification, the PCR product was purified using AM Pure XP beads, and the library was ultimately obtained. The different libraries were pooled according to their effective concentrations and the target amount of data off the machine and then sequenced on the Illumina Nova Seq 6000 platform. The end reading of the 150 bp pairing was generated. The reference genome mm10 and gene model annotation files were downloaded directly from the genome website. An index of the reference genome was built using HISAT2 (v2.0.5), and the paired-end clean reads were aligned to the reference genome using HISAT2 (v2.0.5). Feature counts (v1.5.0-p3) were used to count the number of reads mapped to each gene. The FPKM of each gene was subsequently calculated based on the length of the gene and the number of reads mapped to it. Differential expression analysis between two groups was performed using the DESeq2 R package (1.20.0). The Cluster Profiler R package (3.8.1) was used to test the statistical enrichment of differentially expressed genes in the KEGG pathways. For GSEA, a local version of the GSEA tool (http://www.broadinstitute.org/gsea/index.jsp) and KEGG datasets were used.

### Sample size and sample collection

No statistical methods were used to determine the sample size for in vivo, ex vivo, or in vitro experiments. However, at least three biologically independent samples were used per experimental group and condition. The assignment of samples and experimental animals to their respective groups was performed randomly. Animal procedures (including genotyping and treatments) were performed by investigators unaware of the experimental design.

### Statistical analysis

All the statistical analyses were performed using GraphPad Prism 9 (GraphPad Software). The data are presented as the mean and standard deviation (SD). The difference between two groups was assessed using an unpaired two-tailed Student’s *t-*test for normally distributed or the Mann‒Whitney test for non-normally distributed data. For comparisons involving multiple variables, one-way ANOVA with Dunnett’s multiple comparisons test for normally distributed data or the Kruskal‒Wallis test with Dunn’s multiple comparisons test for non-normally distributed data was used. A *p* value < 0.05 was considered to indicate statistical significance, and significance levels are denoted by asterisks in the figures (*p < 0.05, **p < 0.01 and ***p < 0.001).

## Supplementary information

Supplementary Figures and Tables

## Data Availability

The mRNA sequencing data have been deposited in the NCBI Gene Expression Omnibus (GEO) with the accession code GSE253827. Any additional information required to reanalyze the data reported in this paper is available from the corresponding author upon request.
