# Supplementary material for: Psychological stress-induced systemic corticosterone directly sabotages intestinal stem cells and exacerbates colitis
Source: Cell Discov. 2025 May 13;11:46. doi: 10.1038/s41421-025-00796-y (PMC12075755; doi:10.1038/s41421-025-00796-y)
Supplement: Supplementary file 1 — Supplementary Figures and Tables [file 41421_2025_796_MOESM1_ESM.pdf]

Supplementary Fig. S1

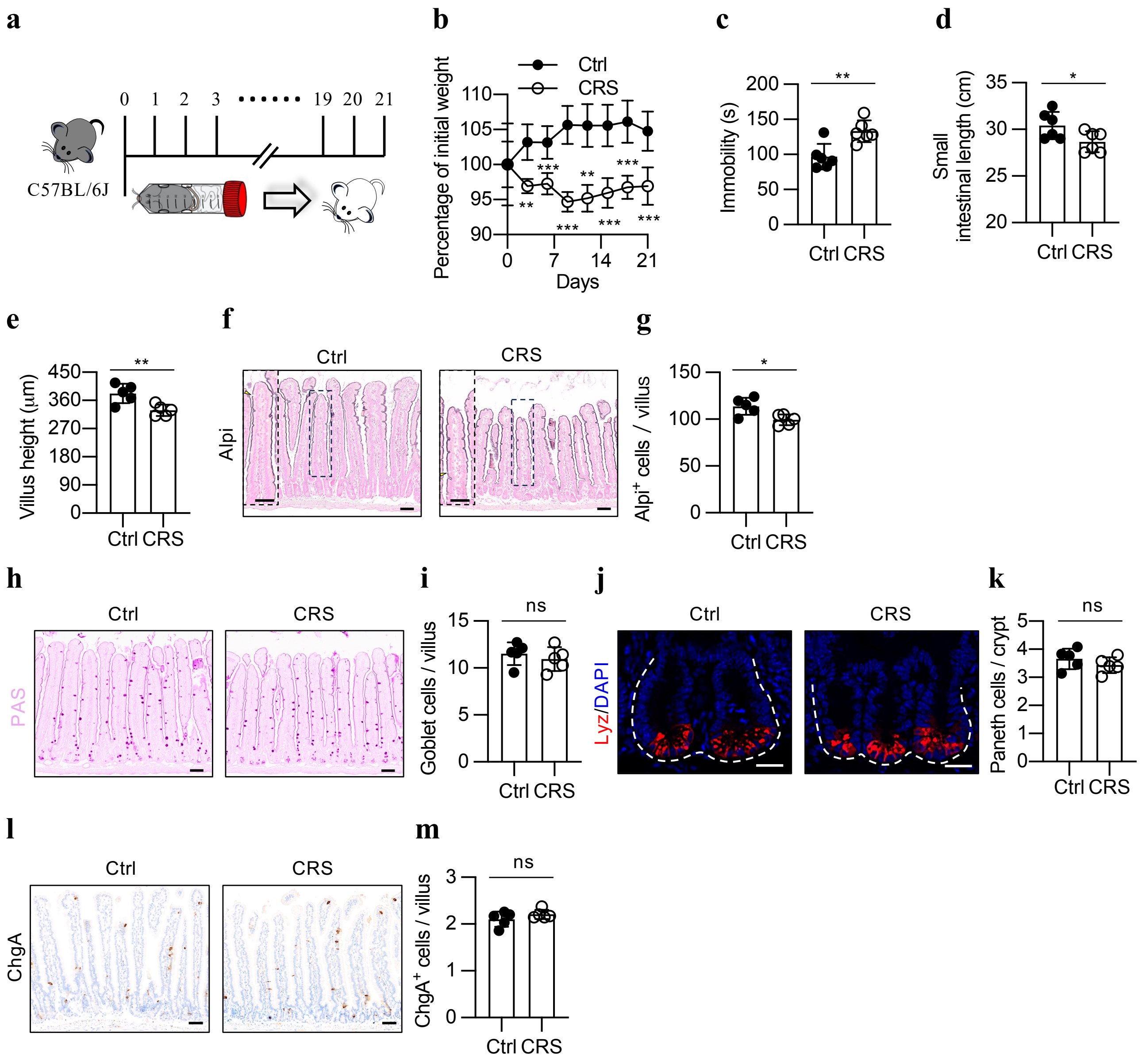

Supplementary Fig. S1 Chronic stress impacts the small intestinal epithelium.

a Schematic of the chronic restraint stress (CRS) mouse model. C57BL/6J mice were subjected to CRS treatment for 3 hours per day over 3 weeks. Control mice were deprived of food and water for the same duration without restraint stress. **b** Change in body weight of control (Ctrl) and CRS-treated mice (n=6) during the CRS treatment period. The body weight was monitored every three days and expressed as a percentage of the initial body weight. **c** Immobility time of the Ctrl and CRS-treated mice in the forced swimming test (FST) (n=6). **d** Small intestine length of Ctrl and CRS-treated mice (n=6). **e** Quantification of villus height in the jejuna of Ctrl and CRS-treated mice (n=5). **f, g** Alpi staining **(f)** and quantification of enterocytes per villus (**g**) in the jejuna of Ctrl and CRS-treated mice (n=5). Yellow arrows pointing to the Alpi-negative epithelial cells. Scale bar: 50 μm. **h, i** PAS staining (**h**) and quantification of mucinous goblet cells per villus (**i**) in the jejuna of Ctrl and CRS-treated mice (n=5). Scale bar: 50 μm. **j, k** Lyz staining (**j**) and quantification of Lyz^+ Paneth cells per crypt (**k**) in the jejuna of Ctrl and CRS-treated mice (n=5). Scale bar: 20 μm. **l, m** ChgA staining (**l**) and quantification of $ChgA^+$ enteroendocrine cells per villus (**m**) in the jejuna of Ctrl and CRS-treated mice (n=5). Scale bar: 50 μm. The data are presented as the mean ± SD. The statistical analysis was performed by an unpaired two-tailed Student's *t* test for normally distributed data or the Mann–Whitney test for non-normally distributed data. ns, $P \geq 0.05$, *$P < 0.05$, **$P < 0.01$.

Supplementary Fig. S2

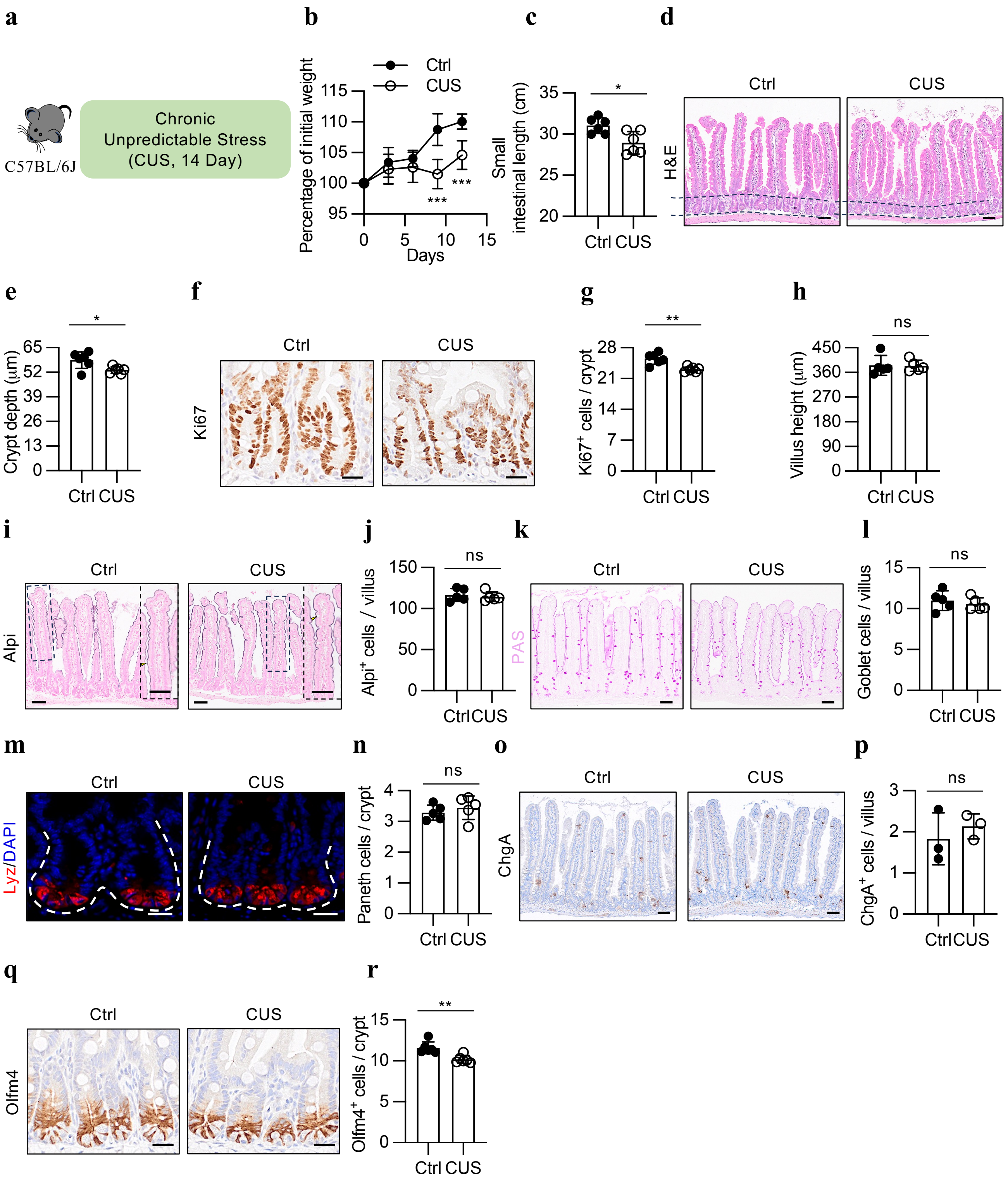

Supplementary Fig. S2 CUS assays validated the detrimental effect of stress on ISCs.

a Schematic of the chronic unpredictable stress (CUS) model in mice. C57BL/6J mice were subjected to CUS treatment daily for 2 weeks. Control mice were similarly deprived of food and water for the same duration without unpredictable stress. **b** Change in body weight of control (Ctrl) and CUS-treated mice (n=6) during the CUS treatment period. The body weight was monitored every three days and expressed as a percentage of the initial body weight. **c** Small intestine length of Ctrl and CUS-treated mice (n=6). **d, e** H&E staining (**d**) and quantification of crypt depth (**e**) in the jejuna of Ctrl and CUS-treated mice (n=6). Scale bar: 50 μm. **f, g** Ki-67 staining (**f**) and quantification of Ki-67^+ cells per crypt (**g**) in the jejuna of Ctrl and CUS-treated mice (n=6). Scale bar: 20 μm. **h** Quantification of villus height in the jejuna of Ctrl and CUS-treated mice (n=5). Scale bar: 50 μm. **i, j** Representative Alpi staining (**i**) and quantification of $Alpi^+$ enterocytes per villus (**j**) in the jejuna of Ctrl and CUS-treated mice (n=5). Yellow arrows pointing to the Alpi-negative epithelial cells. Scale bar: 50 μm. **k, l** PAS staining (**k**) and quantification of mucinous goblet cells per villus (**l**) in the jejuna of Ctrl and CUS-treated mice (n=5). Scale bar: 50 μm. **m, n** Lyz staining (**m**) and quantification of Lyz^+ Paneth cells per crypt (**n**) in the jejuna of Ctrl and CUS-treated mice (n=5). Scale bar: 20 μm. **o, p** ChgA staining (**o**) and quantification of $ChgA^+$ enteroendocrine cells per villus (**p**) in the jejuna of Ctrl and CUS-treated mice (n=3). Scale bar: 50 μm. **q, r** Olfm4 staining (**q**) and quantification of $Olfm4^+$ cells per crypt (**r**) in the jejuna of Ctrl and CUS-treated mice (n=6). Scale bar: 20 μm. The data

are presented as the mean ± SD. The statistical analysis was performed by an unpaired two-tailed Student's *t* test for normally distributed data or the Mann–Whitney test for non-normally distributed data. ns, $P \geq 0.05$, *$P < 0.05$, **$P < 0.01$.

Supplementary Fig. S3

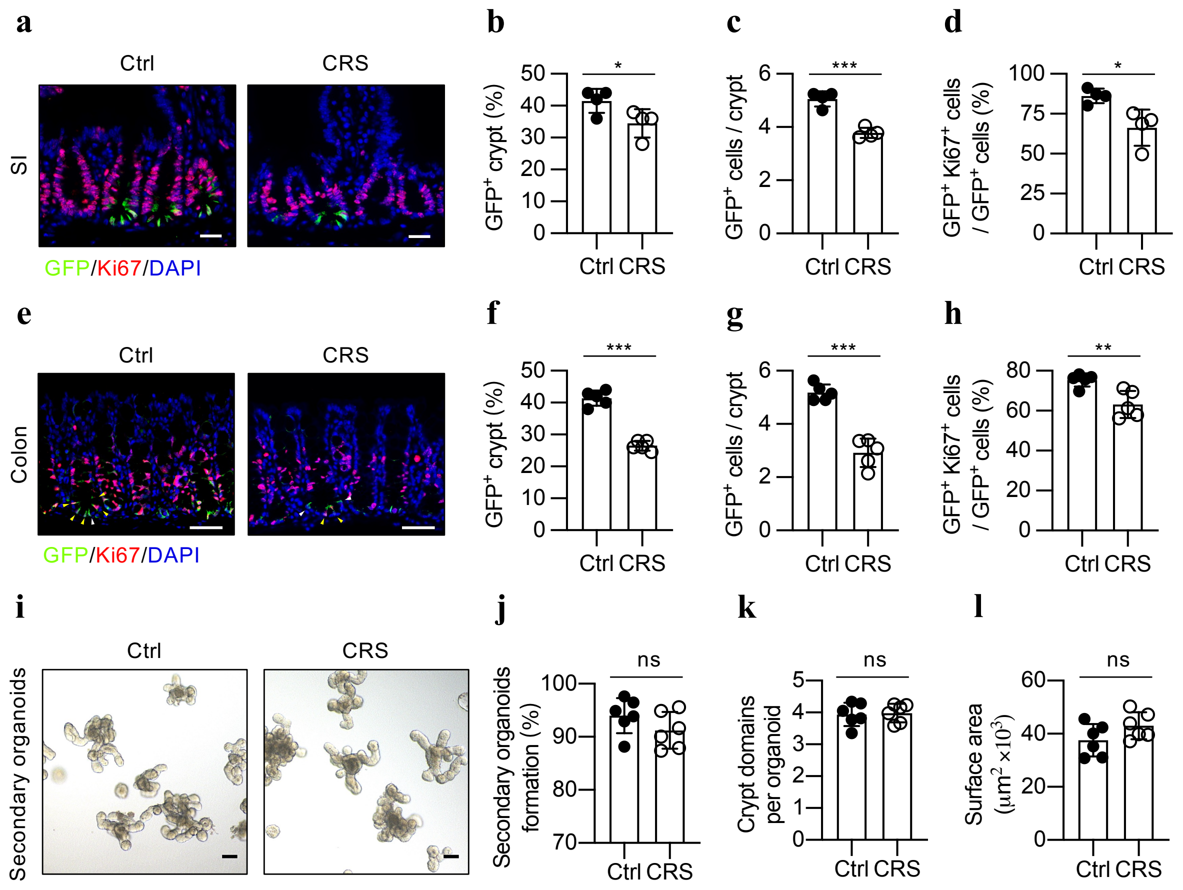

Supplementary Fig. S3 Chronic stress leads to the reduction in the numbers and prolferative activity of colonic stem cells.

a $Lgr5^+$ stem cells were identified with anti-GFP and proliferative state was visualized by Ki-67 staining in the jejuna of control (Ctrl) and CRS-treated *Lgr5-EGFP-IRES-creERT2* mice. SI, small intestine. Scale bar: 20 μm. **b-d** Quantification of percentage of GFP^+ crypt **(b)**, GFP^+ cells per crypt **(c)**, and the proportion of GFP^+ cells expressing Ki-67 **(d)** in the jejuna of Ctrl and CRS-treated *Lgr5-EGFP-IRES-creERT2* mice (n=4). **e** $Lgr5^+$ stem cells were identified with anti-GFP and proliferative state was visualized by Ki-67 staining in the colon of Ctrl and CRS-treated *Lgr5-EGFP-IRES-creERT2* mice. Scale bar: 50 μm. **f-h** Quantification of percentage of GFP^+ crypt **(f)**, GFP^+ cells per crypt **(g)**, and the proportion of GFP^+ cells expressing Ki-67 **(h)** in the colon of Ctrl and CRS-treated *Lgr5-EGFP-IRES-creERT2* mice (n=5). **i** Representative images of day 3 secondary organoids generated from dissociated crypt-derived primary organoids from small intestine of Ctrl and CRS-treated mice. Scale bar: 100 μm. **j–l** Quantification of secondary organoid formation (**j**), bud number (**k**) and surface area (**l**) (n=6). The data are presented as the mean ± SD. The statistical analysis was performed by an unpaired two-tailed Student's *t* test for normally distributed data or the Mann–Whitney test for non-normally distributed data. ns, $P \geq 0.05$, $^{*}P < 0.05$, $^{**}P < 0.01$, $^{***}P < 0.001$.

Supplementary Fig. S4

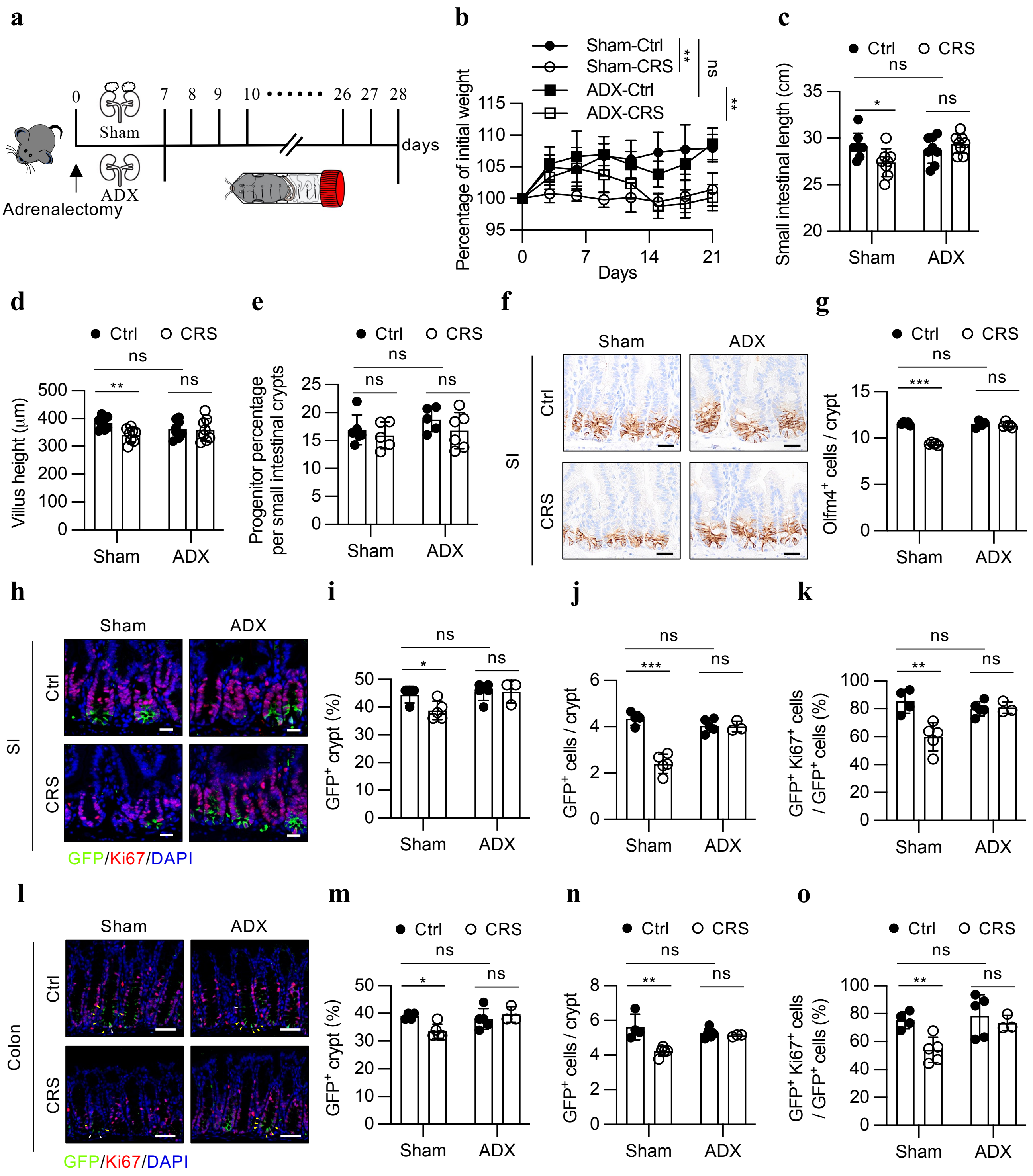

Supplementary Fig. S4 Adrenalectomy counteracts the effect of stress on the intestinal epithelium.

a Schematic of the ADX-CRS paradigm. One week after the operation, a portion of the sham-operated (Sham) mice and the adrenalectomized (ADX) mice were subjected to CRS treatment for 3 weeks. **b** Change in body weight of Sham+Ctrl (n=5), Sham+CRS (n=6), ADX+Ctrl (n=4), and ADX+CRS (n=5) mice during CRS treatment. Body weight was monitored every three days and expressed as a percentage of the initial body weight. **c** Small intestine length of Sham+Ctrl, Sham+CRS, ADX+Ctrl, and ADX+CRS (n=8) mice. **d** Quantification of villus height in the jejuna of Sham+Ctrl, Sham+CRS, ADX+Ctrl, and ADX+CRS (n=8) mice. **e** Quantification of the percentage of progenitor cells (Lgr5-EGFP^{Low}) in the small intestine of Sham+Ctrl (n=6), Sham+CRS (n=5), ADX+Ctrl (n=5) and ADX+CRS *Lgr5-EGFP-IRES-creERT2* mice (n=6) by flow cytometric analysis. **f, g** Olfm4 staining (**f**) and quantification of Olfm4^{+} cells per crypt (**g**) in the jejuna of Sham+Ctrl (n=5), Sham+CRS (n=5), ADX+Ctrl (n=4) and ADX+CRS (n=5) mice. Scale bar: 20 μm. **h** Lgr5^{+} stem cells were identified with anti-GFP, and proliferative state was visualized by Ki-67 staining in the jejuna of Sham+Ctrl, Sham+CRS, ADX+Ctrl and ADX+CRS *Lgr5-EGFP-IRES-creERT2* mice. SI, small intestine. Scale bar: 20 μm. **i-k** Quantification of percentage of GFP^{+} crypt **(i)**, GFP^{+} cells per crypt **(j)**, and the proportion of GFP^{+} cells expressing Ki-67 **(k)** in the jejuna of Sham+Ctrl (n=4), Sham+CRS (n=5), ADX+Ctrl (n=5) and ADX+CRS (n=3) *Lgr5-EGFP-IRES-creERT2* mice. **l** Lgr5^{+} stem cells were identified using anti-GFP, and proliferative state was visualized by Ki-67 staining in the colon of Sham+Ctrl,

Sham+CRS, ADX+Ctrl and ADX+CRS *Lgr5-EGFP-IRES-creERT2* mice. Scale bar: 50 μm. **m-o** Quantification of percentage of GFP^+ crypt **(m)**, GFP^+ cells per crypt **(n)**, and the proportion of GFP^+ cells expressing Ki-67 **(o)** in the colon of Sham+Ctrl (n=4), Sham+CRS (n=5), ADX+Ctrl (n=5) and ADX+CRS (n=3) *Lgr5-EGFP-IRES-creERT2* mice. The data are presented as the mean ± SD. The statistical analysis was performed by an unpaired two-tailed Student's *t* test for normally distributed data or the Mann–Whitney test for non-normally distributed data. ns, $P \geq 0.05$, *$P < 0.05$, **$P < 0.01$, ***$P < 0.001$.

Supplementary Fig. S5

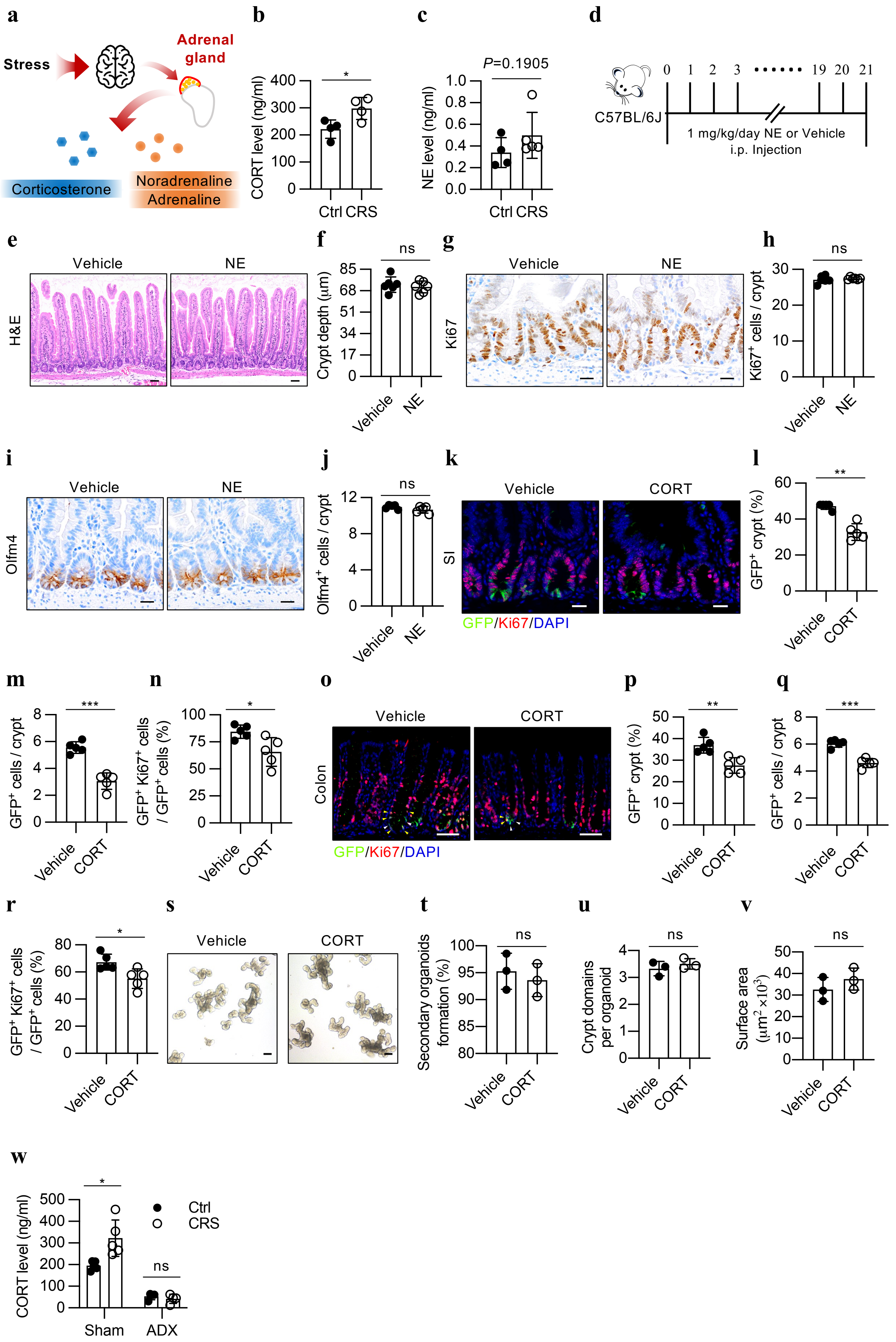

Supplementary Fig. S5 Corticosterone rather than noradrenaline drives the impairment of ISCs.

a Schematic of Corticosterone, Noradrenaline and Adrenaline triggered by stress. **b** Plasma levels of CORT in control (Ctrl) mice and mice subjected to 3 hours of restraint stress (n=4). **c** Plasma levels of NE in Ctrl mice and mice subjected to 3 hours of restraint stress (n=3). NE, noradrenaline. **d** Schematic representation of NE administration to C57BL/6J mice. WT mice were intraperitoneally injected with NE or vehicle (1 mg/kg BW/day) for 3 weeks. **e, f** H&E staining (**e**) and quantification of crypt depth (**f**) in the jejuna of vehicle- and NE-treated mice (n=6). Scale bar: 50 μm. **g, h** Ki-67 staining (**g**) and quantification of $Ki67^+$ cells per crypt (**h**) in the jejuna of vehicle- and NE-treated mice (n=6). Scale bar: 20 μm. **i, j** Olfm4 staining (**i**) and quantification of $Olfm4^+$ cells per crypt (**j**) in the jejuna of vehicle- and NE-treated mice (n=5). Scale bar: 20 μm. **k** $Lgr5^+$ stem cells were identified with anti-GFP, and their proliferative state was visualized using Ki-67 staining in the jejuna of the vehicle- and CORT-treated (5 mg/kg BW/day) *Lgr5-EGFP-IRES-creERT2* mice. SI, small intestine. Scale bar: 20 μm. **l-n** Quantification of the percentage of GFP^+ crypt **(l)**, GFP^+ cells per crypt **(m)**, and the proportion of GFP^+ cells expressing Ki-67 **(n)** in the jejuna of vehicle- and CORT -treated *Lgr5-EGFP-IRES-creERT2* mice (n=5). **o** $Lgr5^+$ stem cells were identified with anti-GFP, and their proliferative state was visualized using Ki-67 staining in the colon of vehicle- and CORT-treated *Lgr5-EGFP-IRES-creERT2* mice. Scale bar: 50 μm. **p-r** Quantification of percentage of GFP^+ crypt **(p)**, GFP^+ cells per crypt **(q)**, and the proportion of GFP^+ cells expressing Ki-67 **(r)** in the colon of vehicle-

and CORT-treated *Lgr5-EGFP-IRES-creERT2* mice (n=5). **s–v** Representative images of day 3 secondary organoids generated from dissociated crypt-derived primary organoids from the vehicle- and CORT-treated groups (**s**) and quantification of secondary organoid formation (**t**), bud number (**u**) and surface area (**v**) (n=3). Scale bar: 100 μm. **w** Plasma levels of CORT in Sham and ADX mice with or without restraint stress treatment (n=4-5). The data are presented as the mean ± SD. The statistical analysis was performed by an unpaired two-tailed Student's *t* test for normally distributed data or the Mann–Whitney test for non-normally distributed data. ns, $P \geq 0.05$, *$P < 0.05$, **$P < 0.01$, ***$P < 0.001$.

Supplementary Fig. S6

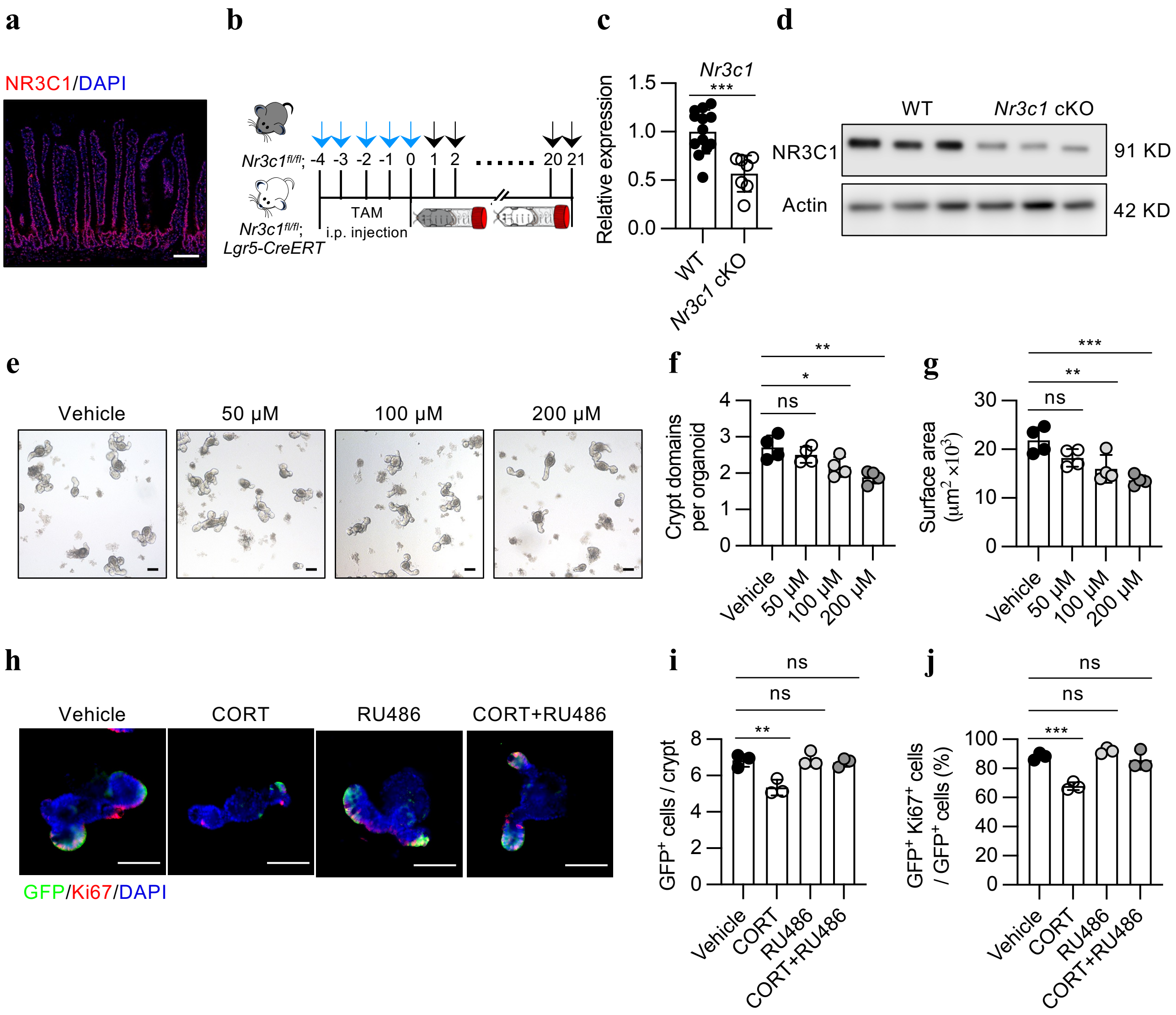

Supplementary Fig. S6 Corticosterone directly impedes ISCs via NR3C1.

a Immunofluorescence staining of NR3C1 in the small intestine of the WT mice. Scale bar: 100 μm. **b** Schematic diagram of CRS treatment in WT and *Nr3c1* cKO mice. After 5 days of tamoxifen (TAM, 2 mg/25 g BW/day) injection, a portion of WT and *Nr3c1* cKO mice were subjected to CRS treatment for 3 weeks. **c** RT–qPCR was used to detect *Nr3c1* expression in small intestinal crypts from WT (n=13) and *Nr3c1* cKO (n=7) mice. **d** Western blotting for NR3C1 expression in small intestinal crypts from WT and *Nr3c1* cKO mice. **e** Representative images of day 4 organoids after *ex vivo* treatment with different concentrations of CORT. Scale bar: 100 μm. **f, g** Quantification of the bud number (**f**) and surface area (**g**) of the organoids (n=4). **h** Representative immunofluorescence images of day 4 organoids from small intestine of *Lgr5-EGFP-IRES-creERT2* mice after *ex vivo* treatment with 200 μM CORT, 5 μM RU486 or 200 μM CORT simultaneously supplemented with 5 μM RU486. $Lgr5^+$ stem cells were identified with anti-GFP, and their proliferative state was visualized by Ki-67 staining. Scale bar: 100 μm. **i,j** Quantification of GFP^+ cells per crypt **(i)**, and the proportion of GFP^+ cells expressing Ki-67 **(j)** in organoids after *ex vivo* treatment with 200 μM CORT, 5 μM RU486 or 200 μM CORT simultaneously supplemented with 5 μM RU486 (n=3). The data are presented as the mean ± SD. Statistical analysis was performed by one-way ANOVA with Dunnett's multiple comparisons test for normally distributed data or Kruskal–Wallis test with Dunn's multiple comparisons test for non-normally distributed data. ns, $P \geq 0.05$, $^{*}P < 0.05$, $^{**}P < 0.01$, $^{***}P < 0.001$.

Supplementary Fig. S7

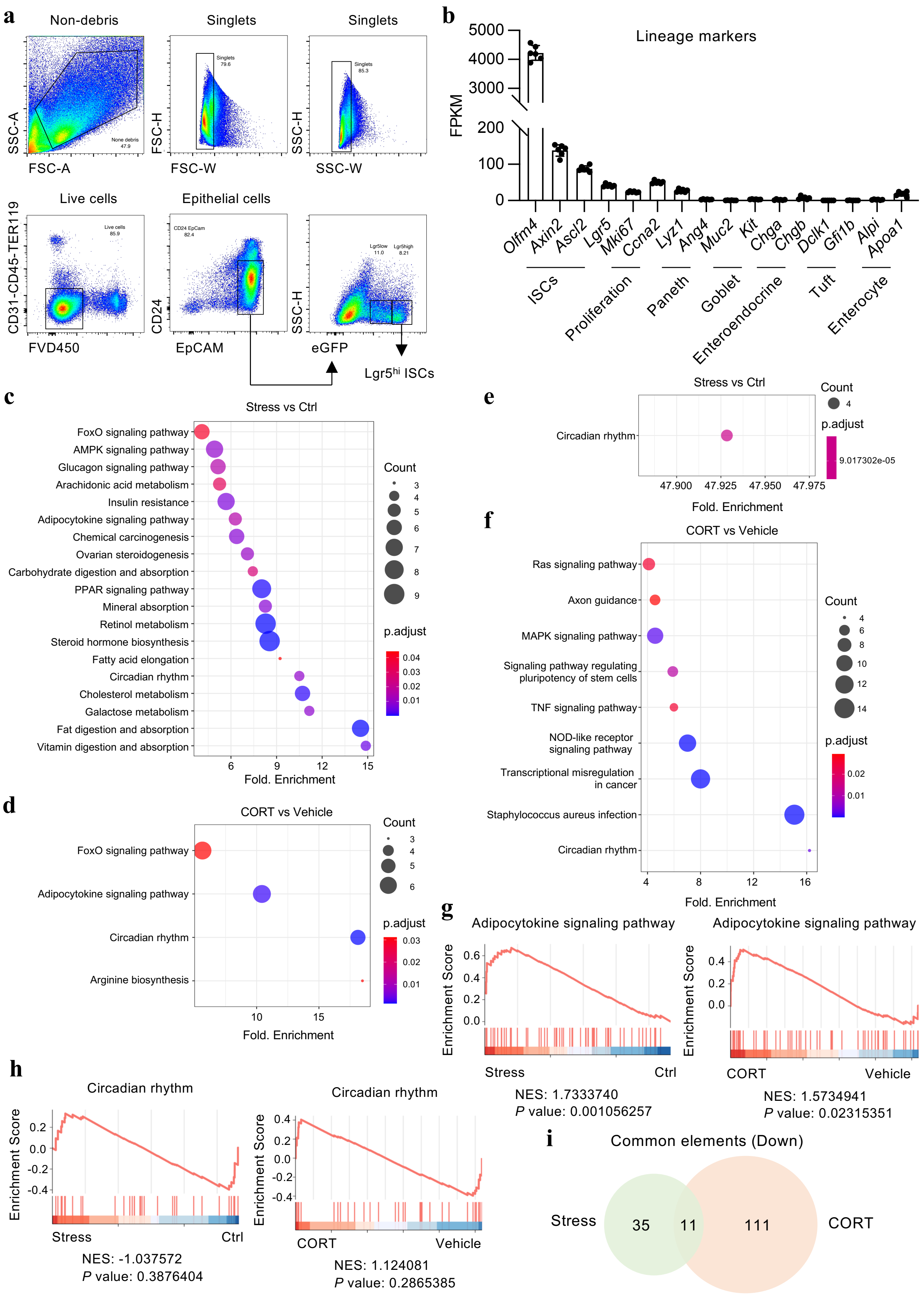

Supplementary Fig. S7 Differential gene expression in ISCs from stress- and corticosterone-treated mice.

a Gating strategy for FACS analysis to isolate Lgr5-EGFP^{high} ISCs from the small intestine of *Lgr5-EGFP-IRES-creERT2* mice for RNA-seq (n=3). **b** Expression levels of different cell type-specific signature genes in FACS-purified Lgr5-EGFP^{high} ISCs. Data are shown as FPKM (fragments per kilobase million). **c, d** KEGG analysis of upregulated genes in ISCs from (**c**) 1-day restraint stress-treated mice (versus Ctrl) and (**d**) 1-day CORT-treated mice (versus vehicle). **e, f** KEGG analysis of downregulated genes in ISCs from (**e**) 1-day restraint stress-treated mice (versus Ctrl) and (**f**) 1-day CORT-treated mice (versus vehicle). **g, h** GSEA of transcriptome profiles showing the enrichment of (**g**) the adipocytokines and (**h**) the circadian rhythm signaling pathway in ISCs from 1-day restraint stress-treated mice (versus Ctrl) and 1-day CORT-treated mice (versus vehicle). **i** Venn diagram showing overlapping downregulated genes in ISCs from 2-day restraint stress-treated mice (versus Ctrl) and 1-day CORT-treated mice (versus vehicle).

Supplementary Fig. S8

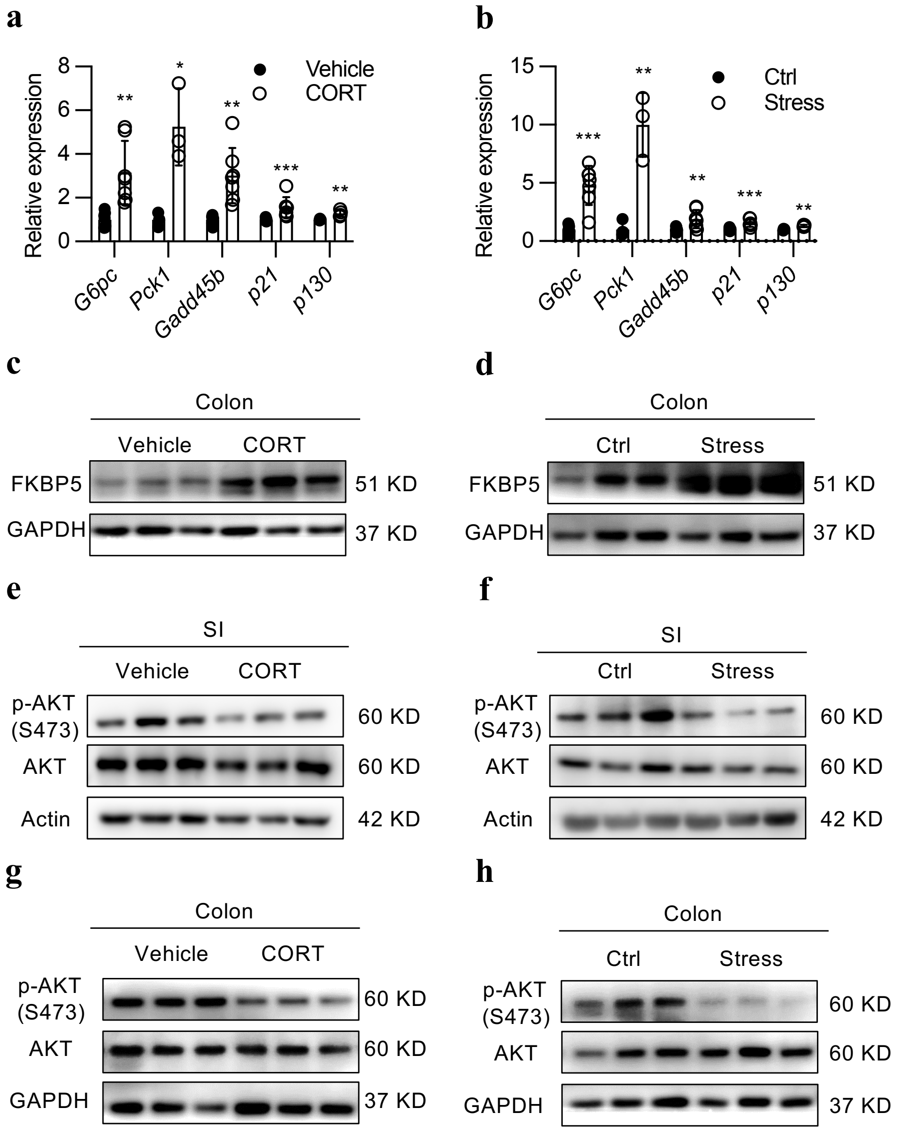

Supplementary Fig. S8 Corticosterone negatively regulates the activation of AKT. **a, b** RT–qPCR analysis of gene expression in the small intestinal crypts from 1-day CORT- (**a**) and 1-day stress-treated (**b)** mice (n=3-7). **c, d** Western blotting for FKBP5 in the colonic crypts of vehicle- and 2-day CORT-treated mice (**c**) and in the colonic crypts of Ctrl and 2-day restraint stress-treated mice (**d**); GAPDH was used as a loading control. **e, f** Western blotting for AKT and pAKT (S473) in the small intestinal crypts of vehicle- and 2-day CORT-treated mice (**e**) and in the small intestinal crypts of Ctrl and 2-day restraint stress-treated mice (**f**); β-actin was used as a loading control. SI, small intestine. **g, h** Western blotting for AKT and pAKT (S473) in the colonic crypts of vehicle- and 2-day CORT-treated mice (**g**) and in the colonic crypts of Ctrl and 2-day restraint stress-treated mice (**h**); GAPDH was used as a loading control. The data are presented as the mean ± SD. The statistical analysis was performed by an unpaired two-tailed Student's *t* test for normally distributed data or the Mann–Whitney test for non-normally distributed data. *$P < 0.05$, **$P < 0.01$, ***$P < 0.001$.

Supplementary Fig. S9

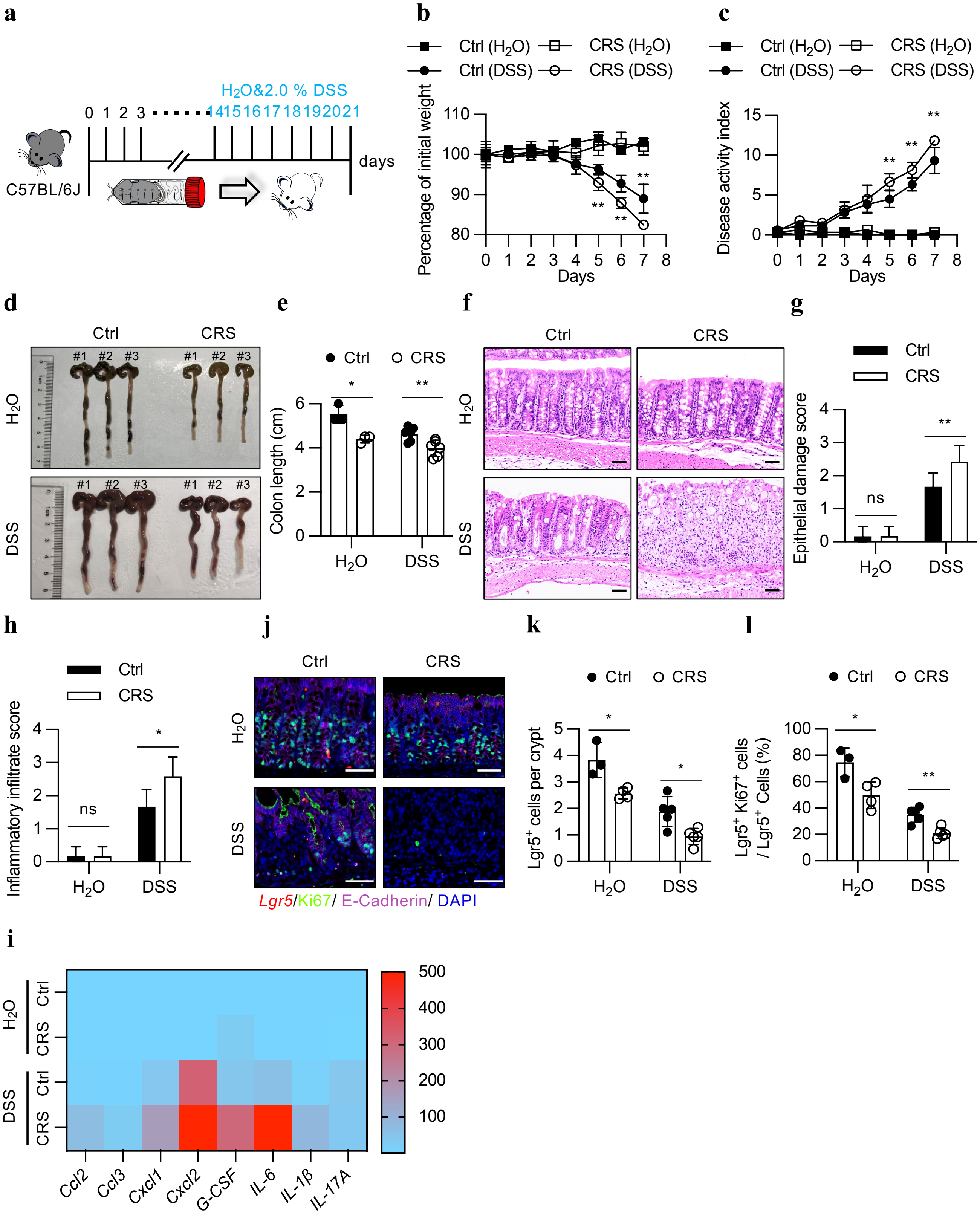

Supplementary Fig. S9 Chronic stress increases the susceptibility to developing colitis.

a Schematic of the dextran sulfate sodium (DSS)-induced colitis model. Water or 2.0 % DSS drinking water was administered during the last week of the three-week CRS treatment. **b, c** Body weight (**b**) and disease activity index (DAI) (**c**) of control (Ctrl) and stress-treated (CRS) mice with (n=6) or without (n=3) 2.0% DSS treatment. Body weight was monitored daily and expressed as a percentage of the initial body weight. **d, e** Representative image of the colon (**d**) and colon length (**e**) of Ctrl and CRS-treated mice with (n=6) or without (n=3) 2.0% DSS treatment. **f–h** Representative HE staining image (**f**), epithelial damage score (**g**), and inflammatory infiltrate score (**h**) of colonic sections from Ctrl and CRS-treated mice with (n=6) or without (n=3) 2.0% DSS treatment. Scale bar: 50 μm. **i** RT–qPCR analysis of cytokine genes in colonic tissue from H_2O+Ctrl(n=3), H_2O+CRS(n=4), DSS+Ctrl (n=6), DSS+CRS (n=8) mice on day 7 during DSS treatment. **j** Representative image of *in situ* hybridization for Lgr5 together with immunofluorescence for Ki-67 and the epithelial marker cadherin (E-cadherin) in the colon of Ctrl and CRS-treated mice with (n=6) or without (n=3) 2.0% DSS treatment. Scale bar: 50 μm. **k-l** Quantification of percentage of $Lgr5^+$ cells per crypt **(k)**, and the proportion of $Lgr5^+$ cells expressing Ki-67 **(l)** in the colon of Ctrl and CRS-treated mice with (n=5) or without (n=3-4) 2.0% DSS treatment. The data are presented as the mean ± SD. The statistical analysis was performed by an unpaired two-tailed Student's *t* test for normally distributed data or the Mann–Whitney test for non-normally distributed data. ns, $P \geq 0.05$, $*P < 0.05$, $**P < 0.01$.

Supplementary Fig. S10

a

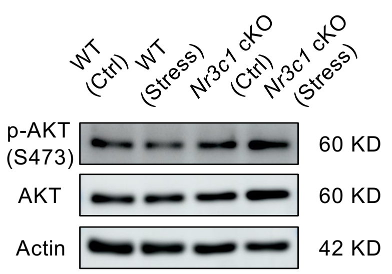

b

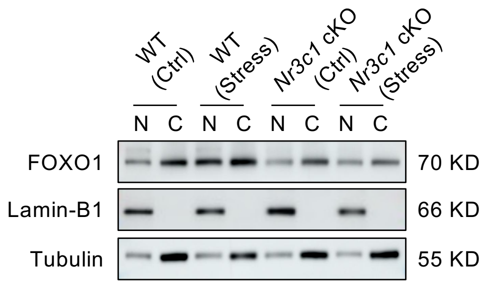

Supplementary Fig. S10 *Nr3c1* deficiency in ISC restores AKT activation inhibited by stress.

a Western blotting for AKT and pAKT (S473) in the small intestinal crypts of Ctrl or 2-day restraint stress-treated WT (*Nr3c1*$^{fl/fl}$) and *Nr3c1* cKO (*Lgr5;Nr3c1*$^{fl/fl}$) mice; β-actin was used as a loading control. **b** Western blotting for FOXO1 in nuclear and cytoplasmic proteins isolated from the small intestinal crypts of the Ctrl or 2-day restraint stress-treated WT (*Nr3c1*$^{fl/fl}$) and *Nr3c1* cKO (*Lgr5;Nr3c1*$^{fl/fl}$) mice; Lamin B1 and tubulin were used as nuclear proteins and cytoplasmic proteins loading controls, respectively.

Graphic abstract

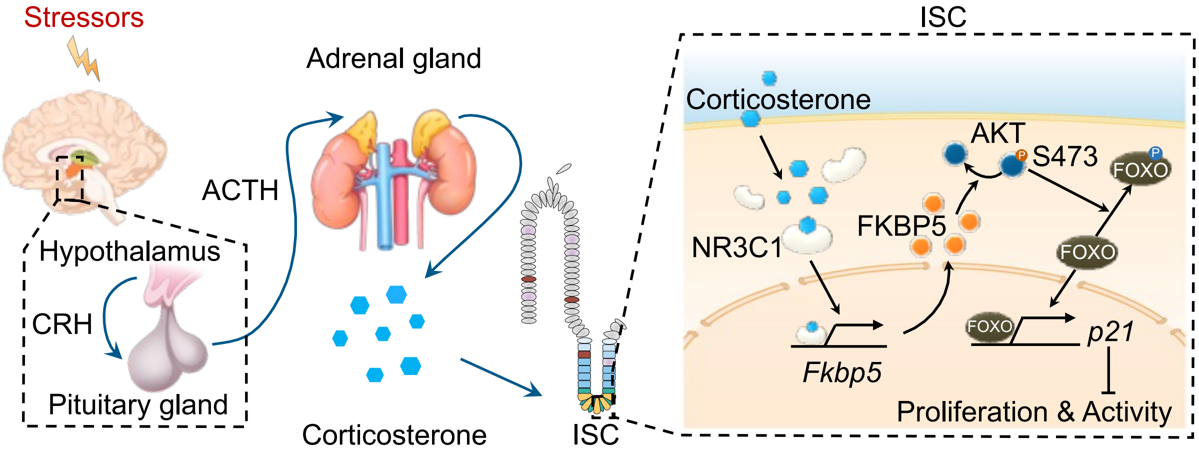

Supplementary Table S1 Genotyping primers

Primer name	Sequence (5' to 3')
Nr3c1 $^{fl/fl}$*-F*	*GCTGCTGCACGGACTGG*
Nr3c1 $^{fl/fl}$*-R*	*CGGCTGCTCTGGAATGTGA*
Lgr5 $^{tm1(cre/ERT2)}$*- Common*	*CTGCTCTCTGCTCCCAGT CT*
Lgr5 $^{tm1(cre/ERT2}$*- Mutant Reverse*	*CTGAACTTGTGGCCGTTTAC*
Lgr5 $^{tm1(cre/ERT2}$*- Wild type Reverse*	*GTCTGGTCAGAATGCCCTTG*

Supplementary Table S2 ChIP-qPCR primers

Primer name	Sequence (5' to 3')
ChIP-Fkbp5 promotor-F	*TTTGCATCTCCGCCTCTTCA*
ChIP-Fkbp5 promotor-R	*TCCTCCATCCCTCTTCTCCG*
ChIP-negative ctrl-F	*GCCAAGTTCAGCTGTGCAAT*
ChIP-negative ctrl-R	*TGCCAGCCACATTCAGAACA*

Supplementary Table S3 RT-qPCR primers

Primer name	Sequence (5' to 3')
m-Nr3c1-F	*TCAGCAGCAGGATCAGAAGC*
m-Nr3c1-R	*TGGACGGAGGAGAACTCACA*
m-Fkbp5-F	*TGAGGGCACCAGTAACAATGG*
m-Fkbp5-R	*CAACATCCCTTTGTAGTGGACAT*
m-Cdkn1a(p21)-F	*CCTGGTGATGTCCGACCTG*
m-Cdkn1a(p21)-R	*CCATGAGCGCATCGCAATC*
m-Ccnd1-F	*GCGTACCCTGACACCAATCTC*
m-Ccnd1-R	*CTCCTCTTCGCACTTCTGCTC*
m-Rbl2(p130)-F	*TCCTTACACGACGGTCTAGTG*
m-Rbl2(p130)-R	*TCCCAGCGGGTAACACGTA*
m-Gadd45b-F	*CAACGCGGTTCAGAAGATGC*
m-Gadd45b-R	*GGTCCACATTCATCAGTTTGGC*
m-Ccl2-F	*AACTCTCACTGAAGCCAGCTCT*
m-Ccl2-R	*CGTTAACTGCATCTGGCTGA*
m-Ccl3-F	*TGAAACCAGCAGCCTTTGCTC*
m-Ccl3-R	*AGGCATTCAGTTCCAGGTCAGTG*
m-Cxcl1-F	*CAATGAGCTGCGCTGTCAGTG*
m-Cxcl1-R	*CTTGGGGACACCTTTTAGCATC*
m-Cxcl2-F	*CCTGGTTCAGAAAATCATCCA*
m-Cxcl2-R	*CTTCCGTTGAGGGACAGC*
m-G-CSF-F	*ATGGCTCAACTTTCTGCCCAG*
m-G-CSF-R	*CTGACAGTGACCAGGGGAAC*

Primer name	***Sequence (5′ to 3′)***
m-IL-6-F	*CACGGCCTTCCCTACTTCAC*
m-IL-6-R	*TGCAAGTGCATCATCGTTGT*
m-IL-1β-F	*TGTGGCTGTGGAGAAGCTGT*
m-IL-1β-R	*CAGCTCATATGGGTCCGAGA*
m-IL-17A-F	*GCTCCAGAAGGCCCTCAG*
m-IL-17A-R	*CTTTCCCTCCGCATTGACA*
m-Actb-F	*GAGCGCAAGTACTCTGTGTG*
m-Actb-R	*CGGACTCATCGTACTCCTG*
